# A Nitric Oxide–Based STING Agonist Nanoadjuvant With ICD‐Derived Membrane Antigens Unleashes Robust Antitumor Immunity

**DOI:** 10.1002/exp2.70238

**Published:** 2026-10-11

**Authors:** Shuyu Xu, Yang Song, Tianjiao Zhang, Yan Wang, Mengxin Wang, Xinxin Xie, Qingfeng Chen, Xiaolong Liang

**Affiliations:** ^1^ Beijing Key Laboratory of Ultrasound Targeted Drug Delivery and Frontier Imaging Technology, Department of Ultrasound Peking University Third Hospital Beijing P. R. China; ^2^ Department of Ultrasound Medicine, Beijing Chaoyang Hospital Capital Medical University Chaoyang Beijing China; ^3^ Department of Ultrasound The First Affiliated Hospital of China Medical University Shenyang China; ^4^ Institute of Molecular and Cell Biology Agency For Science, Technology and Research Singapore Singapore; ^5^ State Key Laboratory of Vascular Homeostasis and Remodeling, Beijing Key Laboratory of Interdisciplinary Research in Gastrointestinal Oncology (BLGO) Peking University Third Hospital Beijing P. R. China

**Keywords:** gas vaccine adjuvant, nanovaccine, nitric oxide, tumor vaccine

## Abstract

Despite significant advances in nanovaccine development, their efficacy remains hindered by poor antigen presentation, limited antigen repertoire, and suboptimal T cell priming. Immune adjuvants such as polyinosinic‐polycytidylic acid (POLY(I:C)) exert antitumor effects by promoting dendritic cell (DC) maturation and stimulating innate immunity, but their efficacy is limited by poor antigen presenting cell (APC) uptake and weak CD8^+^ T cell responses. Here, we propose a novel adjuvant strategy using nitric oxide (NO), a bioactive gaseous molecule, to upregulate MHC‐I and enhance DC cross‐presentation, thereby synergistically improving the adjuvant efficacy of POLY(I:C). We developed a nanovaccine, Im@NOnanoPL, by co‐encapsulating a preassembled complex of DOTAP, NOC‐18, and POLY(I:C) into a PLGA shell, which was further modified with DSPE‐PEG_2000_ and camouflaged with immunogenic cell death (ICD)–derived tumor membranes for broad‐spectrum tumor antigen coverage. Im@NOnanoPL elicited robust and durable CD8^+^ T cell responses, conferring near‐complete prophylactic protection and, in combination with αPD‐1 therapy, eradicating established tumors while inducing long‐term immune memory. Im@NOnanoPL constitutes a novel vaccine platform that integrates a gas‐based adjuvant with broad‐spectrum antigens, exhibiting strong translational potential.

## Graphical abstract

Schematic illustration of the synergistic antitumor mechanism of Im@NOnanoPL combined with αPD‐1 therapy. The Im@NOnanoPL nanovaccine targets lymph nodes via ICD‐derived tumor cell membranes, delivering personalized tumor antigens. It releases NO gas to enhance MHC‐I expression and activate the STING pathway, thereby potentiating anti‐tumor immunity in combination with αPD‐1 therapy.

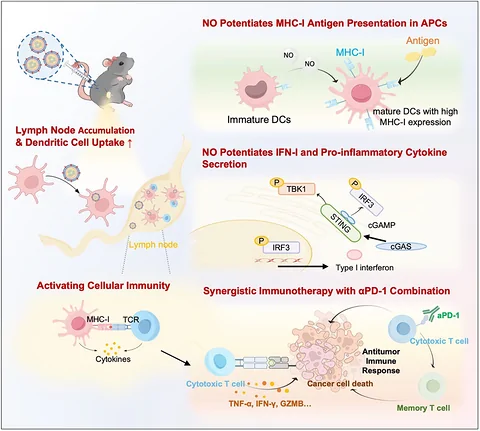

## 1 Introduction

Recently, cancer vaccines have emerged as a promising strategy in tumor immunotherapy due to their ability to induce long‐lasting, tumor‐specific immune responses [1, 2]. A fundamental mechanism underlying the efficacy of these vaccines lies in their capacity to stimulate CTLs, which can directly recognize and eliminate malignant cells [3, 4]. Several cancer vaccines have already been approved or are under clinical investigation. For instance, Sipuleucel‐T (Provenge) [5], an FDA‐approved dendritic cell‐based vaccine, has shown survival benefit in metastatic castration‐resistant prostate cancer. Additionally, peptide‐based vaccines targeting tumor‐associated antigens such as NY‐ESO‐1 [6, 7], WT1 [8, 9], and HPV E6/E7 [10, 11] proteins have shown promising therapeutic efficacy in clinical trials. More recently, neoantigen‐based personalized vaccines [12, 13] and mRNA cancer vaccines (e.g., mRNA‐4157 [14, 15], BNT122 [16, 17]) have advanced into early‐phase trials with promising immunological and clinical responses [18, 19, 20]. Despite these developments, the overall efficacy of many cancer vaccines remains limited due to factors such as poor antigen presentation, immunosuppressive tumor microenvironment, and inadequate T cell priming [21].

Given that effective CTL priming critically depends on efficient antigen cross‐presentation, recent studies have focused on improving adjuvant designs to strengthen this process and thereby enhance vaccine efficacy [22]. POLY(I:C), a synthetic analog of double‐stranded RNA, is a classical agonist of TLR3 and MDA5 and has been widely employed as a vaccine adjuvant [23, 24, 25, 26, 27, 28]. It promotes DC maturation and enhances innate immune activation. However, despite these advantages, its ability to induce potent and durable CD8^+^ T cell responses remains limited, particularly following subcutaneous administration where efficient lymph node priming is required. Enhancing DC cross‐presentation is therefore a critical strategy for improving vaccine efficacy, not only to overcome the limited immunogenicity associated with restricted antigen repertoires but also to achieve robust MHC‐I upregulation, thereby enabling effective activation of cytotoxic T lymphocytes.

In addition to immunogenicity, another critical barrier to vaccine efficacy is limited delivery efficiency. Poor delivery hinders the accumulation of vaccine components in lymph nodes and reduces uptake by DCs, the primary site for antigen presentation and T cell priming [29]. Within the lymph node microenvironment, antigen‐presenting cells particularly DCs capture and process antigens, upregulate co‐stimulatory molecules, and present peptides via MHC‐I to naïve CD8^+^ T cells. Given this critical immunological function, the targeted delivery of antigens and immunostimulatory adjuvants to lymph nodes is essential for inducing potent and sustained antitumor immunity [30, 31, 32]. Accordingly, effective nanovaccines should hold capability to accumulate in lymph nodes, protect antigens from degradation, and enable efficient uptake by DCs [33, 34, 35]. Rationally designed nanovaccine platforms typically comprise tumor antigens, immunostimulatory adjuvants, and biocompatible carriers, forming a multifunctional delivery system that enhances immune priming [36, 37]. Despite these advancements, most current cancer vaccines rely on a few well‐characterized, fixed epitopes as antigens. This limited antigen diversity can reduce efficacy in heterogeneous tumors and increase the risk of immune escape.

Nitric oxide, an endogenously produced bioactive gaseous molecule, possesses high biocompatibility and the ability to freely diffuse and has demonstrated therapeutic effects in a variety of diseases [38, 39, 40]. In this study, we found that NO can induce type I interferon production and upregulate MHC‐I expression on antigen‐presenting cells (APCs). We hypothesize that the combination of NO and POLY(I:C) could more efficiently initiate antigen cross‐presentation by APCs in lymph nodes, representing a novel adjuvant strategy for cancer vaccination. The efficacy of POLY(I:C) strongly depends on its uptake by DCs, whereas the gaseous and freely diffusible nature of NO allows it to exert its effects independent of DC uptake efficiency. As a novel adjuvant combination, these two components are expected to synergistically enhance DC‐mediated cross‐presentation, leading to robust and sustained activation of CD8^+^ T cells. The cationic lipid DOTAP, NOC‐18 (NO donor), and POLY(I:C) were first assembled into a complex, which was subsequently co‐encapsulated within a PLGA shell. The nanoparticle surface was further modified with DSPE‐PEG2000 to enhance biocompatibility. This design enables stable delivery of POLY(I:C) and sustained release of NO, while integrating the biocompatibility of lipids with the controlled‐release properties of polymers, thereby achieving prolonged and effective drug delivery. Tumor cell membranes derived from immunogenic cell death (ICD) were selected as the source of cancer vaccine antigens. Unlike single or peptide antigens, ICD membranes provide a broad repertoire of tumor‐associated membrane antigens, offering broad epitope coverage to overcome tumor heterogeneity. In addition, damage‐associated molecular patterns (DAMPs) on the surface of ICD membranes enhance DC antigen uptake. Simple physical encapsulation of antigens together with the adjuvants resulted in the formation of the Im@NOnanoPL vaccine platform (Scheme 1).

**SCHEME 1:**
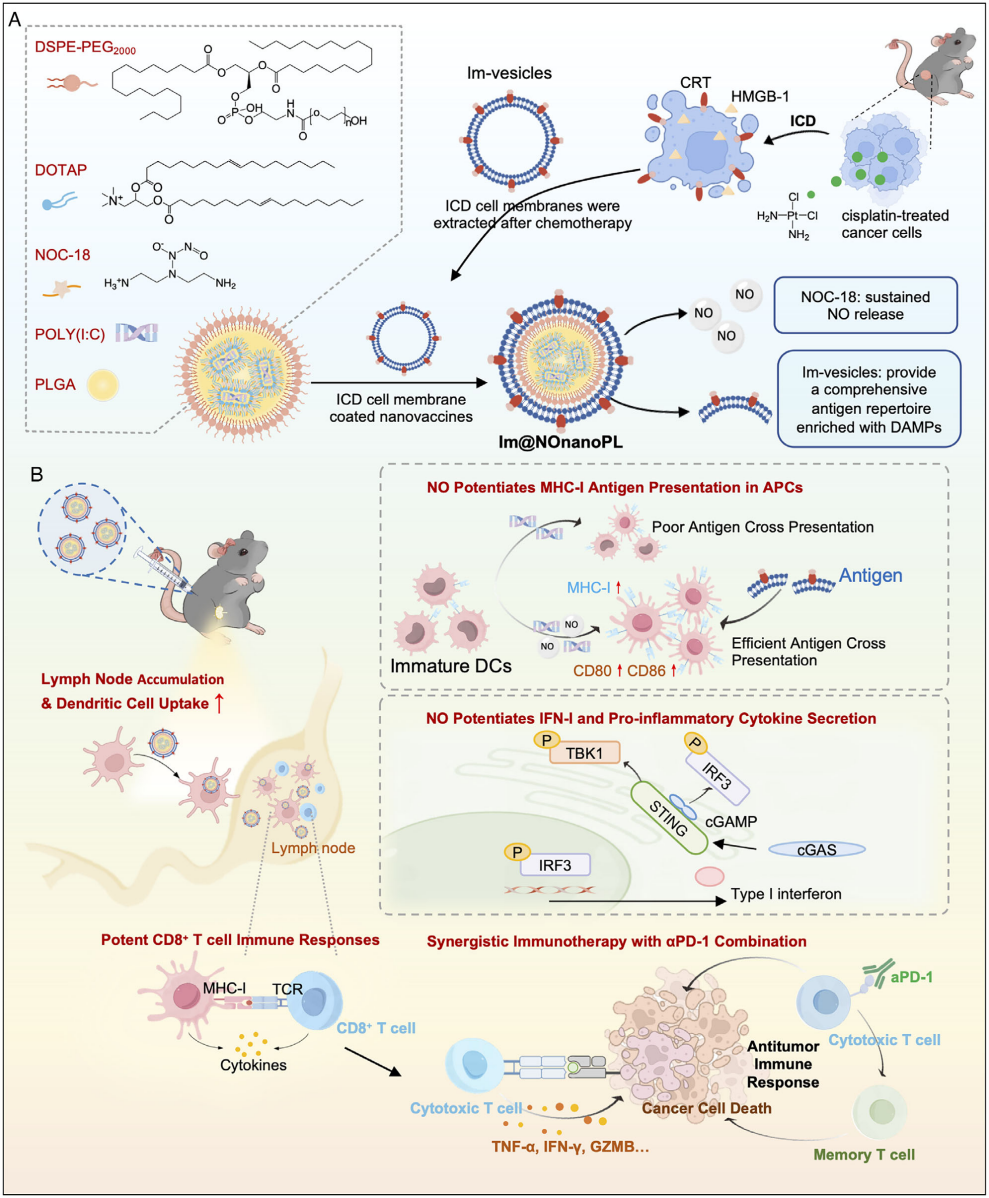
(A) Preparation of Im@NOnanoPL Vaccine; (B) Schematic illustration of the synergistic antitumor mechanism of Im@NOnanoPL combined with αPD‐1 therapy.

## 2 Results and Discussion

### 2.1 NO Enhances Antigen Presentation and Immunostimulatory Functions of DCs

MHC‐I molecules play a central role in presenting intracellular or processed antigens to CD8^+^ T cells and are therefore critical for effective cytotoxic T lymphocyte (CTL) responses. We first investigated whether NO could enhance antigen presentation in dendritic cells (DCs). To directly assess antigen‐specific presentation, bone marrow‐derived dendritic cells (BMDCs) were co‐cultured with the OVA_257–264_ (SIINFEKL) peptide in the presence of NOC‐18, an NO donor, and the resulting MHC‐I_OVA_ complex was quantified by flow cytometry (Figure 1a). Treatment with NOC‐18 (150 µM) significantly increased MHC‐I_OVA_ expression at both 12 and 24 h compared with the untreated control (Figure 1b), indicating that NO enhances the presentation of the OVA‐derived peptide by BMDCs. We next examined whether NO could broadly enhance MHC‐I expression in DCs beyond the presentation of a defined antigenic peptide. NOC‐18 treatment significantly increased surface MHC‐I expression on BMDCs, with a nearly two‐fold increase observed at 24 h compared with untreated cells (Figure 1c). This effect was concentration dependent over the range of 25–150 µM, whereas a slight reduction in MHC‐I expression was observed at 200 µM, potentially reflecting reduced cellular viability at the higher concentration (Supplementary Figure S1). Consistent with these findings, NOC‐18 also markedly increased MHC‐I expression in bone marrow‐derived macrophages (BMDMs), accompanied by reduced CD206 expression (Supplementary Figures S2 and S3). Furthermore, GSNO, an alternative NO donor, similarly increased both MHC‐I_OVA_ and total MHC‐I expression in BMDCs (Supplementary Figures S4 and S5), supporting the association of these effects with NO signaling rather than a donor‐specific effect.

**FIGURE 1:**
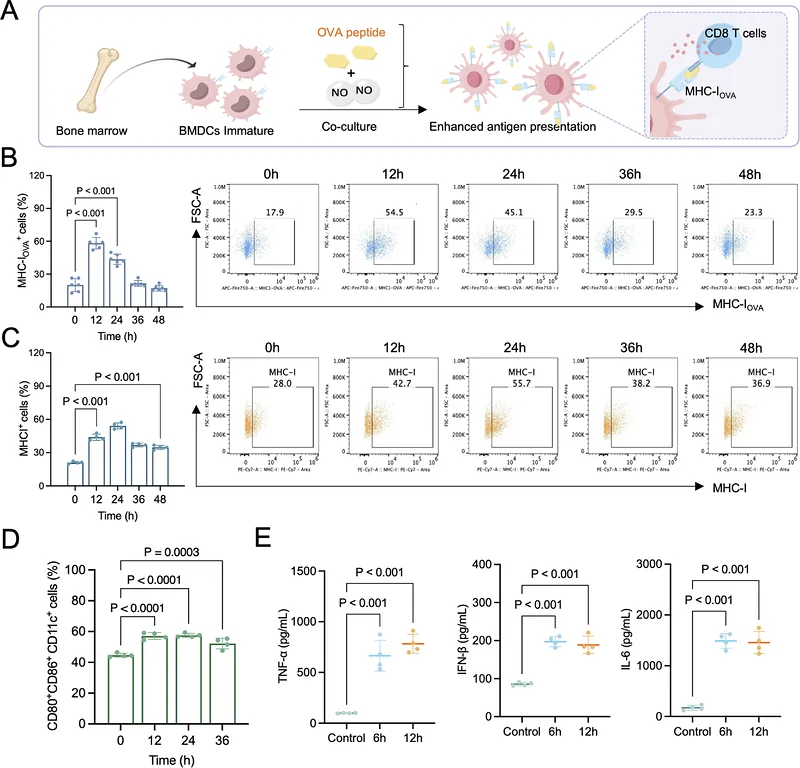
Nitric oxide potentiates DC antigen presentation and immune activation. (A) Schematic representation of NO potentiates DC antigen presentation and immune activation. (B) Evaluation of MHC‐I_OVA_ complex levels in BMDCs after 0, 12, 24, 36, and 48 h incubation with NOC‐18 (150 µM), as detected by flow cytometry (*n* = 6). (C) Evaluation of MHC‐I levels in BMDCs after 0, 12, 24, 36, and 48 h incubation with NOC‐18 (150 µM), as detected by flow cytometry (*n* = 4). (D) Evaluation of CD80^+^ CD86^+^ DCs under 0, 12, 24, and 36 h incubation with NOC‐18 (150 µM) (*n* = 4). (E) Levels of pro‐inflammatory cytokines TNF‐𝛼, IFN‐𝛽 and IL‐6, in the supernatant of BMDCs following different treatments. Results are presented as mean ± SD (*n* = 4). Statistical analysis was performed using the one‐way ANOVA with Tukey's multiple comparisons test.

Because effective antigen presentation is closely associated with DC activation and maturation, we next examined the expression of the co‐stimulatory molecules CD80 and CD86. NOC‐18 treatment increased the proportion of CD80^+^CD86^+^ BMDCs, indicating enhanced DC maturation (Figure 1d). In parallel, NOC‐18 treatment increased the secretion of the inflammatory cytokines TNF‐α, IFN‐β, and IL‐6 into the culture supernatant (Figure 1e). Together, these findings demonstrate that NO enhances both antigen‐presenting capacity and immunostimulatory activity in DCs, characterized by increased antigen‐MHC‐I complex formation, elevated MHC‐I expression, DC maturation, and inflammatory cytokine production. These properties support the use of NO as an immunomodulatory component for enhancing the activity of cancer vaccine platforms.

### 2.2 Design and Characterization of Im@NOnanoPL Nanovaccine

To enhance the lymph node targeting capability of NO gas vaccines and meet the requirement for highly personalized antigen matching, we developed cisplatin‐treated tumor cell membrane vesicles (Figure 2a). The chemotherapeutic agent cisplatin induces ICD in tumor cells, leading to the release of tumor antigens and immune‐stimulatory signals that can further activate immune responses. The ICD‐derived cell membranes preserve all surface antigens of the original tumor cells, including unidentified neoantigens, thereby overcoming the limitations of artificial antigen selection while ensuring precise antigen matching for personalized medicine [41, 42, 43]. These chemotherapy‐induced ICD membranes display DAMPs that promote antigen uptake by APCs and improve the lymph node targeting efficiency of the nanovaccine. The nanovesicle structure not only protects antigens from degradation and prolongs immune stimulation but also enhances lymph node targeting and APC uptake through its nano‐architecture. To confirm cisplatin‐induced ICD, we performed confocal microscopy analysis showing upregulated calreticulin (CRT) expression on the membrane of treated MC38 colorectal cancer cells (Figure 2b), accompanied by increased extracellular ATP levels (Figure 2c). The ICD tumor cell membranes (Im‐vesicles) were isolated through ultrasonication and physical extrusion. Flow cytometric analysis demonstrated significantly higher CRT expression on Im‐vesicles compared to membranes from untreated tumor cells (m‐vesicles) (Figure 2d). We then further constructed the nanovaccine using poly (lactic‐co‐glycolic acid) (PLGA) as the carrier to co‐encapsulate the vaccine adjuvant POLY(I:C) and NO donor NOC‐18. In the preparation process, POLY(I:C), the cationic lipid DOTAP, and NOC‐18 were first thoroughly mixed and then added dropwise into the PLGA solution. The incorporation of PLGA significantly improved the stability and enabled sustained release of NOC‐18. The resulting PLGA nanoparticles were subsequently modified with DSPE‐PEG_2000_ to enhance their hydrophilicity, followed by physical extrusion with ICD cell membranes to form the final nanovaccine, designated as Im@NOnanoPL. The loading of the functional components in Im@NOnanoPL was quantitatively evaluated using component‐specific assays. POLY(I:C) encapsulation was determined by a RiboGreen RNA assay following nanoparticle disassembly, yielding a high encapsulation efficiency of 98.8%, indicating effective electrostatic complexation and polymeric confinement. The NO donor NOC‐18 was quantified by measuring the residual unencapsulated fraction in the supernatant after nanoparticle formation using the Griess assay, and the encapsulation efficiency was calculated to be 91.5%.

**FIGURE 2:**
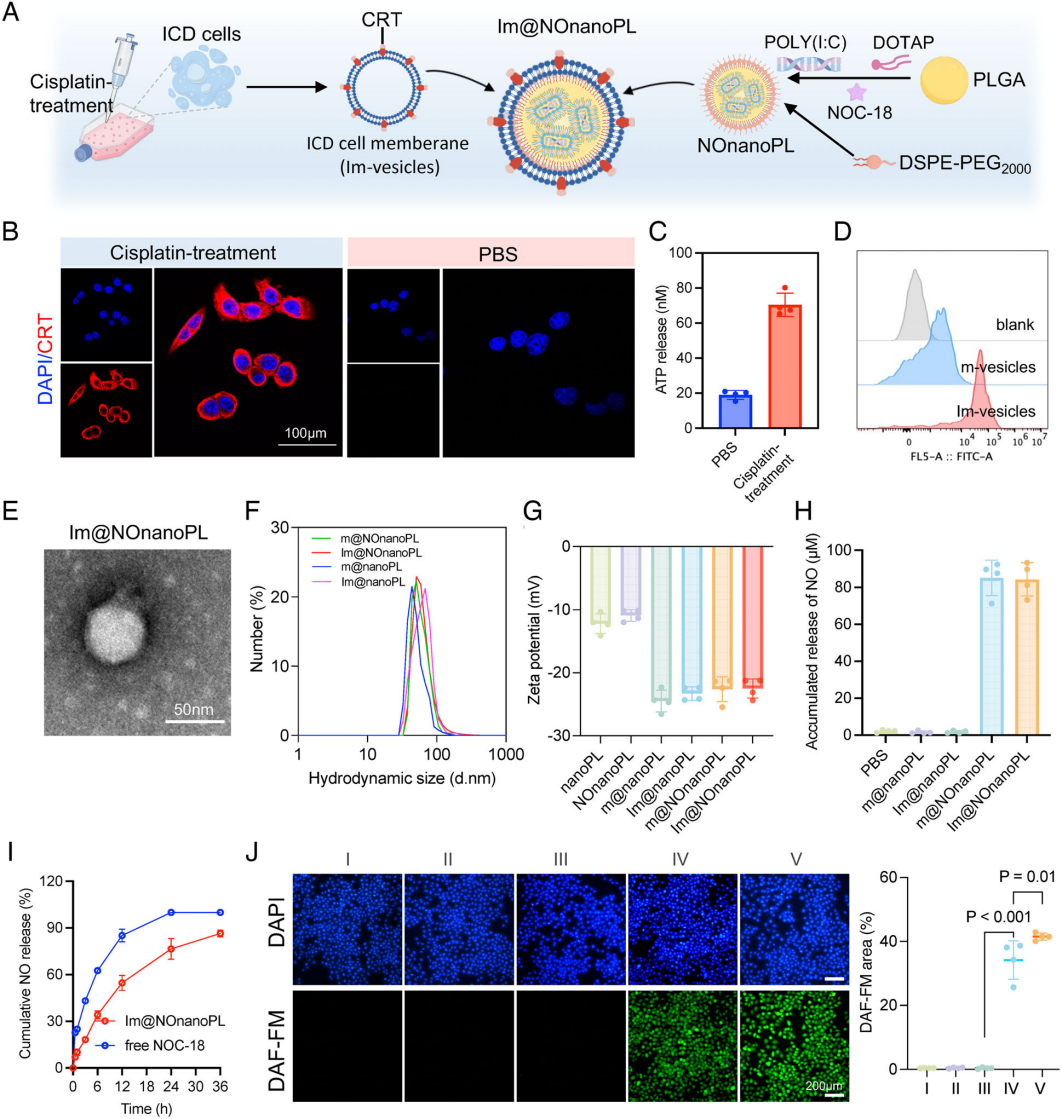
Characterizations of Im@NOnanoPL and the ability to release NO. (A) Schematic preparation of the Im@NOnanoPL system. (B–D) Characterizations of cisplatin induced ICD: (B) CRT exposure analyzed by confocal laser scanning microscopy, scale bar 100 µm, and (C) ATP secretion. (D) CRT exposure on the surface of m‐vesicles or Im‐vesicles measured by flow cytometry. The blank control represents Im‐vesicles without anti‐CRT antibody staining, used to define background fluorescence and gating. (E) TEM image of Im@NOnanoPL, scale bar 50 nm. (F) Particle size distribution curve of Im@NOnanoPL and other formulations. (G) Zeta potential distribution of nanoparticles with/without NO loading and membrane coating. (H) Cumulative release of NO from PBS, Im@NOnanoPL and other formulations in PBS (pH 7.4, 37°C). (I) Time‐course NO release profiles of free NOC‐18 and Im@NOnanoPL in PBS (pH 7.4, 37°C). (J) NO generation of Im@NOnanoPL and other formulations in DC2.4 cells by fluorescence imaging using the DAF‐FM DA kit and quantitative analysis of fluorescence area. Scale bar: 200 µm. (I: PBS, II: m@nanoPL, III: Im@nanoPL, IV: m@NOnanoPL, V:Im@NOnanoPL). Results are presented as mean ± SD (*n* = 4). Statistical analysis was performed using the one‐way ANOVA with Tukey's multiple comparisons test.

We systematically prepared four nanovaccine formulations for comparative studies: (1) m@nanoPL—nanoparticles coated with untreated cell membranes without NOC‐18; (2) Im@nanoPL—nanoparticles coated with ICD membranes without NOC‐18; (3) m@NOnanoPL—nanoparticles coated with untreated cell membranes containing NOC‐18; and (4) Im@NOnanoPL nanoparticles coated with ICD membranes containing NOC‐18. Comprehensive characterization of these nanovaccines included TEM imaging (Figure 2e and Supplementary Figure S6), which revealed consistent core‐shell spherical structures with similar sizes (approximately 45 nm). DLS measurements (Figure 2f) confirmed uniform hydrodynamic diameters ranging from 45–55 nm (PDI < 0.3) for all formulations. The zeta potential shifted from approximately −12 mV before membrane coating to below −20 mV after coating, providing additional evidence for successful membrane modification (Figure 2g).

Quantification of NO loading using the Griess reagent demonstrated that Im@NOnanoPL and m@NOnanoPL released approximately 80 µM NO over 48 h after removal of unencapsulated NOC‐18 (Figure 2h). NOC‐18 is a diazeniumdiolate‐based NO donor that spontaneously decomposes under physiological, proton‐rich conditions to release NO [44, 45]. We first evaluated NO release under room temperature conditions to assess donor stability prior to administration. Free NOC‐18 released approximately 62.5% of its total NO within 24 h, whereas Im@NOnanoPL released only ∼28.5% over the same period, indicating that PLGA encapsulation markedly suppresses premature NO decomposition and improves donor stability during handling and storage. Under physiological conditions (37°C), kinetic studies further demonstrated that PLGA encapsulation significantly prolonged NO release compared with free NOC‐18, with Im@NOnanoPL exhibiting a sustained release profile and an apparent half‐life of approximately 10.5 h (Figure 2i). Together, these results confirm that Im@NOnanoPL provides both enhanced stability under non‐physiological conditions and controlled, delayed NO release under physiological conditions. At the cellular level, we evaluated DC2.4 cell uptake and subsequent NO production by incubating the cells with either Im@NOnanoPL or m@NOnanoPL for 12 h followed by DAF‐FM DA staining (Figure 2j). Both formulations induced detectable NO production, with Im@NOnanoPL showing stronger fluorescence signals, likely attributable to enhanced cellular uptake mediated by the ICD membranes. In summary, we have successfully developed an innovative ICD membrane‐coated nanovaccine platform that simultaneously delivers personalized tumor antigens, immune adjuvants, and NO donors. This integrated system combines the advantages of lymph node targeting, antigen protection, and controlled NO release to potentially enhance anti‐tumor immune responses.

### 2.3 Immune Effects of Im@NOnanoPL In Vitro

In vitro biocompatibility of Im@NOnanoPL was first evaluated in DC2.4 cells. As shown in Supplementary Figure S7a, Im@NOnanoPL exhibited no detectable cytotoxicity over a concentration range of 0–150 µM, as assessed by the CCK‐8 assay. Consistently, Calcein‐AM/propidium iodide staining revealed no apparent membrane damage in DC2.4 cells treated with Im@NOnanoPL at 100 µM (Supplementary Figure S7b). Moreover, Rhodamine 123 staining showed that treatment with 100 µM Im@NOnanoPL did not disrupt mitochondrial membrane potential, indicating an absence of mitochondrial dysfunction (Supplementary Figure S7c). Collectively, these results demonstrate the favorable in vitro biocompatibility of Im@NOnanoPL at concentrations used for subsequent experiments. The internalization efficiency of nanoparticles and subsequent antigen processing and presentation by APCs represent the first and crucial step in initiating adaptive immune responses. We first investigated DC uptake of nanoparticles by labeling the nanovaccines with fluorescent Cy5‐NHS through co‐incubation with cell membranes. Flow cytometry analysis revealed time‐dependent nanoparticle uptake by DCs, with Im@NOnanoPL showing consistently stronger fluorescence intensity than m@NOnanoPL at all time points (Figure 3a,b), indicating enhanced uptake mediated by ICD membranes. Confocal microscopy images (Supplementary Figure S8) further confirmed that all nanovaccine formulations (m@nanoPL, Im@nanoPL, m@NOnanoPL, Im@NOnanoPL) colocalized with lysosomes in BMDCs at 2 h, which is the pathway for antigen processing by DCs. The classical cross‐presentation pathway involves antigen escape from lysosomes to cytosol, proteasomal degradation into peptides, TAP‐mediated transport to ER, and subsequent loading onto MHC‐I molecules for presentation to CD8^+^ T cells. Lysosomal escape represents another critical step determining antigen presentation efficiency. Confocal images (Figure 3c) demonstrated that Cy5‐labeled Im@NOnanoPL colocalized with lysosomes at 2 h but achieved lysosomal escape by 6 h. We then evaluated antigen presentation using ICD membrane‐coated Im@NOnanoPL, which significantly enhanced MHC‐I_OVA_ complex levels and MHC‐I expression in BMDCs (Supplementary Figure S9 and Figure 3d), indicating superior antigen presentation capacity and CD8^+^ T cell activation potential.

**FIGURE 3:**
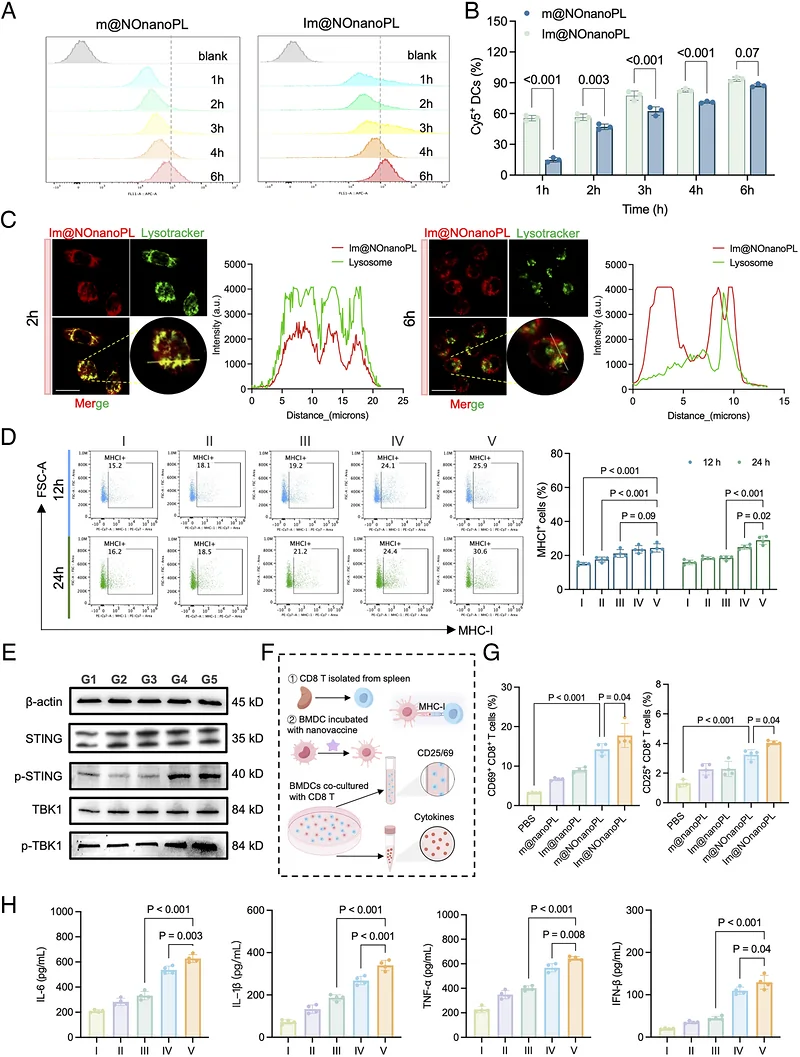
In vitro properties of Im@NOnanoPL nanovaccine. (A) Flow cytometric analysis of DC2.4 cells co‐incubated with Im@NOnanoPL‐Cy5 and m@NOnanoPL‐Cy5 after different durations, respectively. (B) Percentage of Cy5^+^ DC2.4 cells after incubation with Im@NOnanoPL‐Cy5 and m@NOnanoPL‐Cy5. (C) Confocal microscopic images of DC2.4 cells after incubating with Im@NOnanoPL‐Cy5 for 2 h and 6 h. The lysosomes stained with Lysotracker Green (green) and amide‐coupled Cy5 labeling for stable cell membrane vesicles surface modification, Scale bar: 10 µm. (D) Evaluation of MHC‐I levels in BMDCs after 12 and 24 h incubation with Im@NOnanoPL and other formulations (NOC‐18: 100 µM), as detected by flow cytometry (*n* = 4). (I: PBS, II: m@nanoPL, III: Im@nanoPL, IV: m@NOnanoPL, V: Im@NOnanoPL). (E) Western blot analysis of cGAS‐STING pathway‐related proteins expression in BMDCs treated by Im@NOnanoPL and other formulations (NOC‐18: 100 µM) in 12 h (G1: PBS, G2: m@nanoPL, G3: Im@nanoPL, G4: m@NOnanoPL, G5: Im@NOnanoPL). (F) Experimental design for T cell activation and cytokine release analysis. BMDCs were treated with different formulations for 12 h, followed by co‐culture with CD3^+^ T cells at a 1:10 ratio (BMDCs: T cells). The CD3^+^ T cells were isolated from splenocytes of MC38 tumor‐bearing mice. (G) Percentages of CD69^+^ or CD25^+^ in CD3^+^ CD8^+^ T cells after 24 h co‐culture, as detected using flow cytometry (*n* = 4). (H) Cytokines released by T cells and DCs from different groups after 24 h co‐culture, including IL‐6, IL‐1β, TNF‐α, IFN‐β. Results are presented as mean ± SD (*n* = 4). Statistical analysis was performed using the one‐way ANOVA with Tukey's multiple comparisons test.

Furthermore, Im@NOnanoPL effectively activated the STING pathway in BMDCs, as evidenced by elevated p‐TBK1 and p‐STING expression after 12 h co‐culture (Figure 3e). For T cell activation assays, nanovaccine‐treated BMDCs were co‐cultured with CD8^+^ T cells (1:10 ratio) for 24 h (Figure 3f). Under non‐cytotoxic conditions, Im@NOnanoPL treatment effectively activated STING signaling in DC2.4 cells, as evidenced by increased phosphorylation of STING and TBK1. Notably, this activation occurred without detectable mitochondrial dysfunction or cellular damage, arguing against DNA damage–driven cGAS activation as the primary mechanism. These findings demonstrate that Im@NOnanoPL enhances STING‐TBK1‐IRF3 signaling in DCs, together with increased antigen presentation and T cell activation. Flow cytometry analysis of CD69 and CD25 expression (Figure 3g and Supplementary Figure S10) revealed the strongest CD8^+^ T cell activation by Im@NOnanoPL, with m@NOnanoPL showing intermediate effects. ELISA analysis of supernatant cytokines (IL‐6, IL‐1β, TNF‐α, and IFN‐β) confirmed that Im@NOnanoPL stimulated the highest cytokine production, consistent with robust CD8^+^ T cell activation (Figure 3h). These results demonstrate that Im@NOnanoPL as a tumor vaccine exhibits excellent antigen presentation capacity and strong potential for CD8^+^ T cell activation.

### 2.4 Im@NOnanoPL Enhances Lymph Node Targeting and DC Uptake In Vivo

The efficient delivery of antigens to lymph nodes and their uptake by APCs are crucial for developing tumor vaccines based on antigen‐specific CTLs. To elucidate how Im@NOnanoPL achieves efficient lymph node targeting, we examined the distribution and cellular uptake of Cy5.5‐labeled nanoparticles in draining lymph nodes. Our data demonstrate that Im@NOnanoPL is efficiently internalized by both CD103^+^CD11c^+^ migratory DCs and CD8^+^CD11c^+^ resident DCs, two key dendritic cell subsets responsible for antigen cross‐presentation (Supplementary Figure S11). These results support that Im@NOnanoPL accesses lymph nodes through two complementary routes: (i) uptake by local subcutaneous DCs followed by migration to draining lymph nodes, and (ii) direct lymphatic drainage of nanoparticles into lymph nodes, where they are captured by resident DCs.

To evaluate the in vivo lymph node targeting and DCs uptake capability of the prepared nanovaccines, Cy5‐labeled Im@NOnanoPL or m@NOnanoPL was injected into the right gluteal region of mice. At 12, 24, or 36 h post‐injection, mice were euthanized, and the proximal and distal draining lymph nodes were harvested for fluorescence imaging using an in vivo imaging system (IVIS). As shown in Figure 4a,b, the fluorescence intensity in both proximal and distal draining lymph nodes was significantly higher in the Im@NOnanoPL group than in the m@NOnanoPL group. To further assess biodistribution, major organs, including the heart, liver, spleen, lung, and kidney, were collected at 6 h post‐injection and subjected to ex vivo IVIS imaging. Although moderate fluorescence signals were observed in the liver and kidney, the signal intensity in draining lymph nodes was substantially higher than that in all major organs (Supplementary Figure S12), indicating preferential lymph node accumulation of Im@NOnanoPL. This enhanced lymph node accumulation may result from a combination of factors, including the optimal nanoparticle size (<100 nm), which facilitates lymphatic drainage, and the ICD membrane coating, which promotes uptake by subcutaneous DCs and subsequent transport to draining lymph nodes. Further validation by flow cytometry confirmed the uptake of nanovaccines by DCs in lymph nodes. Consistent with the IVIS results, the Cy5 fluorescence intensity in lymph node DCs was significantly higher in the Im@NOnanoPL group at all time points (12, 24, and 36 h) compared to the m@NOnanoPL group (Figure 4c–f). These findings further demonstrate that Im@NOnanoPL exhibits enhanced lymph node targeting and DC uptake efficiency.

**FIGURE 4:**
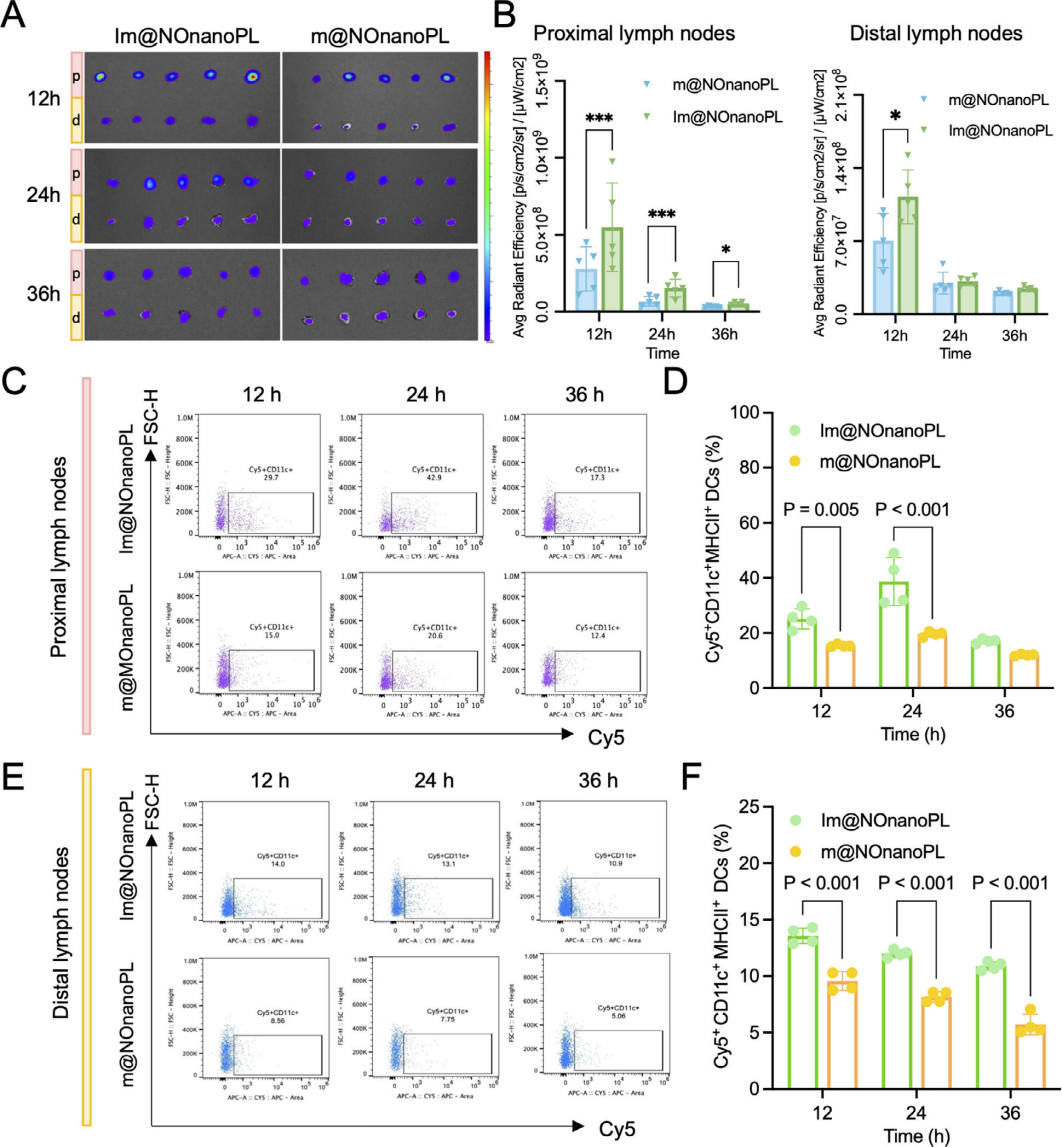
Evaluation of nanovaccine property for lymphatic targeting and DC uptake. (A and B) Ex vivo fluorescence imaging (A) and fluorescent intensities (B) of the lymph nodes harvested from the mice at 12, 24, 36 h after inoculation of Im@NOnanoPL‐Cy5 with m@NOnanoPL or Im@NOnanoPL (*n* = 5 mice per group). (p: proximal lymph nodes, d: distal lymph nodes). Statistical analysis was performed using two‐way ANOVA followed by Tukey's multiple comparisons test. (C and D) Flow cytometry analysis of DC uptake of nanovaccines in proximal lymph nodes: (C) Representative flow cytometry plots showing nanoparticle‐positive DC populations (gated on CD11c^+^ Cy5^+^ cells). (D) Quantitative analysis of nanovaccine uptake by DCs, *n* = 4. (E and F) Flow cytometry analysis of DC uptake of nanovaccines in distal lymph nodes: (E) Representative flow cytometry plots showing nanoparticle‐positive DC populations (gated on CD11c^+^ Cy5^+^ cells). (F) Quantitative analysis of nanovaccine uptake by DCs, *n* = 4. Statistical analysis was performed using two‐way ANOVA followed by Tukey's multiple comparisons test.

### 2.5 Im@NOnanoPL Elicited Effective Immune Responses In Vivo

Having established that Im@NOnanoPL induced strong immune responses in vitro and demonstrated excellent lymph node targeting, we proceeded to investigate its ability to elicit antitumor immune responses in vivo. Healthy C57BL/6N mice were divided into five groups (I: PBS, II: m@nanoPL, III: Im@nanoPL, IV: m@NOnanoPL, V: Im@NOnanoPL) and received subcutaneous vaccinations on days 1 and 7, with lymph nodes and spleens harvested on day 14 (Figure 5a). Initial evaluation of lymph node immune responses revealed that vaccinated mice exhibited lymph node enlargement compared to untreated controls, with the Im@NOnanoPL group showing the most pronounced enlargement (Supplementary Figure S13). This observation was consistent with increased immune activation and prompted further characterization of lymph node immune responses. Subsequent analysis of MHC‐I_OVA_ complex levels and MHC‐I expression in DCs (Figure 5b–d and Supplementary Figure S14) confirmed that Im@NOnanoPL effectively enhanced antigen presentation capacity, consistent with our in vitro findings. The Im@NOnanoPL group demonstrated a significantly elevated frequency of MHC‐I positive DCs (MHC‐I^+^ CD11c^+^), showing a 2.5‐fold increase compared to PBS‐treated controls. Examination of DC maturation demonstrated that the percentage of mature DCs (CD80^+^CD86^+^CD11c^+^) in the Im@NOnanoPL group was significantly higher than other groups (Figure 5e), again aligning with in vitro observations. Flow cytometric analysis of T cells in lymph nodes showed that Im@NOnanoPL treatment resulted in marked activation of effector functions in both CD8^+^ and CD4^+^ T cell populations (Figure 5f,g, and Supplementary Figures S15–S18), with the proportion of GZMB‐positive CD8^+^ T cells increasing from approximately 7.0% in the PBS group to about 24.8%, and IFN‐γ secretion rising from around 1.9% to approximately 8.1%. Additionally, we observed significantly elevated serum concentrations of cytokines (including IFN‐β, IL‐1β, IL‐6, and TNF‐α) following Im@NOnanoPL administration (Figure 5h), indicating systemic immune activation.

**FIGURE 5:**
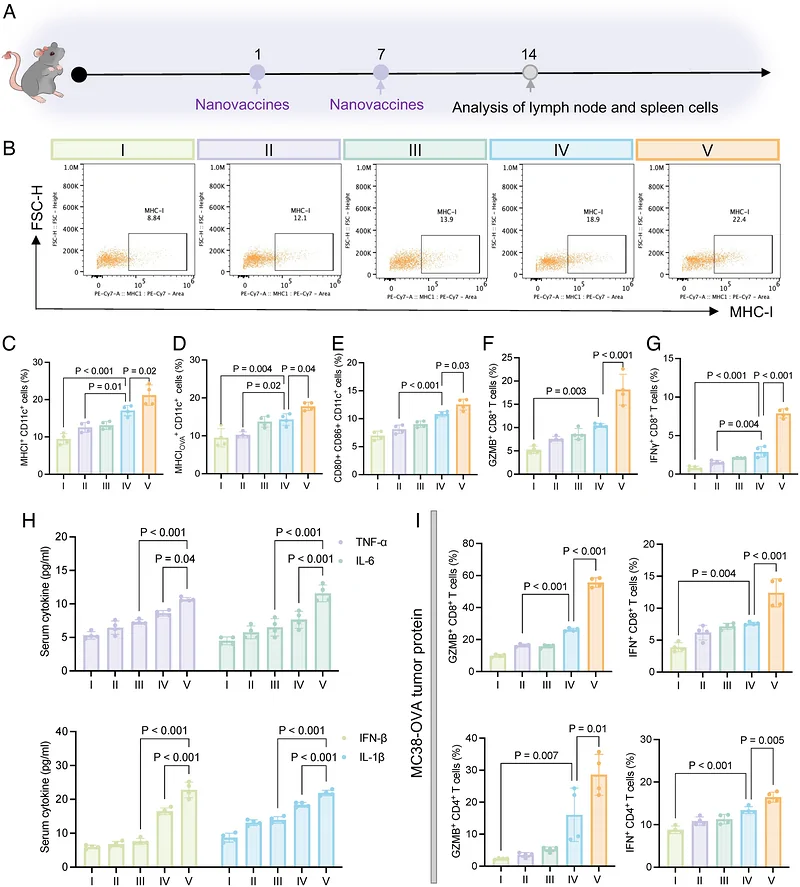
Im@NOnanoPL nanovaccine promoted the activation of immune responses in the spleen and lymph nodes. (A) Schematic representation of mice receiving the indicated treatments. (B–C) Flow cytometry analysis of MHC‐I expression on DCs in lymph nodes under different treatments (*n* = 4). (D) Evaluation of MHC‐I_OVA_ complex levels in DCs in lymph nodes under different treatments (*n* = 4). (E) Maturation of DCs in lymph nodes as measured by the proportions of CD80^+^ CD86^+^ among CD11c^+^ DCs (*n* = 4). Quantification of immune responses of CD8^+^ T cells in the lymph nodes as measured by the percentage of (F–G) GZMB ^+^ among (F) CD8^+^ T cells, and (G) IFN‐γ^+^ among CD8^+^ T cells (*n* = 4). (H) Detection of pro‐inflammatory cytokine concentrations in serum by ELISA kit, including IFN‐β, IL‐1 β, IL‐6, and TNF‐a (*n* = 4). Statistical analysis was performed using one‐way ANOVA followed by Tukey's multiple comparisons test. (I) Flow cytometry analysis of IFN‐γ and granzyme B expression in CD4^+^ and CD8^+^ T cells from vaccinated mice after MC38‐OVA cell protein stimulation (*n* = 4). Statistical analysis was performed using the one‐way ANOVA with Tukey's multiple comparisons test. (I: PBS, II: m@nanoPL, III: Im@nanoPL, IV: m@NOnanoPL,V: Im@NOnanoPL).

Evaluation of adaptive immune responses in the spleen, another critical site of immune activity, involved analysis of splenic single‐cell suspensions after 24‐h stimulation with either phorbol myristate acetate (PMA) and ionomycin (nonspecific stimulation) or MC38 cell proteins (antigen‐specific stimulation) to assess key effector molecules of T cell‐mediated immune killing. Following PMA and ionomycin stimulation, flow cytometry revealed that the Im@NOnanoPL group exhibited the highest frequencies of IFN‐γ^+^ and GZMB^+^ T cells among all groups (Supplementary Figure S19). Similar results were obtained with antigen‐specific stimulation using MC38‐OVA tumor cell proteins, where the Im@NOnanoPL group showed approximately 5.5‐fold more GZMB^+^CD8^+^ T cells and 3‐fold more IFN‐γ^+^CD8^+^ T cells compared to the PBS control group (Figure 5i). These collective findings demonstrate that the Im@NOnanoPL nanovaccine effectively induces comprehensive immune responses in vivo.

### 2.6 Prophylactic Antitumor Efficacy of IM@NOnanoPL

Building upon our previous findings demonstrating lymph node targeting of Im@NOnanoPL and immune activation properties, we next investigated its potential for preventing B16 melanoma lung metastasis. The nanovaccine was personalized using membranes from cisplatin‐treated B16‐OVA tumor cells to match the metastatic model. Mice received a three‐dose vaccination via subcutaneous injection on days 1, 7, and 14 (Figure 6a) and were challenged with 2 × 10^5^ B16‐OVA cells via tail vein injection 7 days after the last dose. Lung metastasis was evaluated on day 35.

**FIGURE 6:**
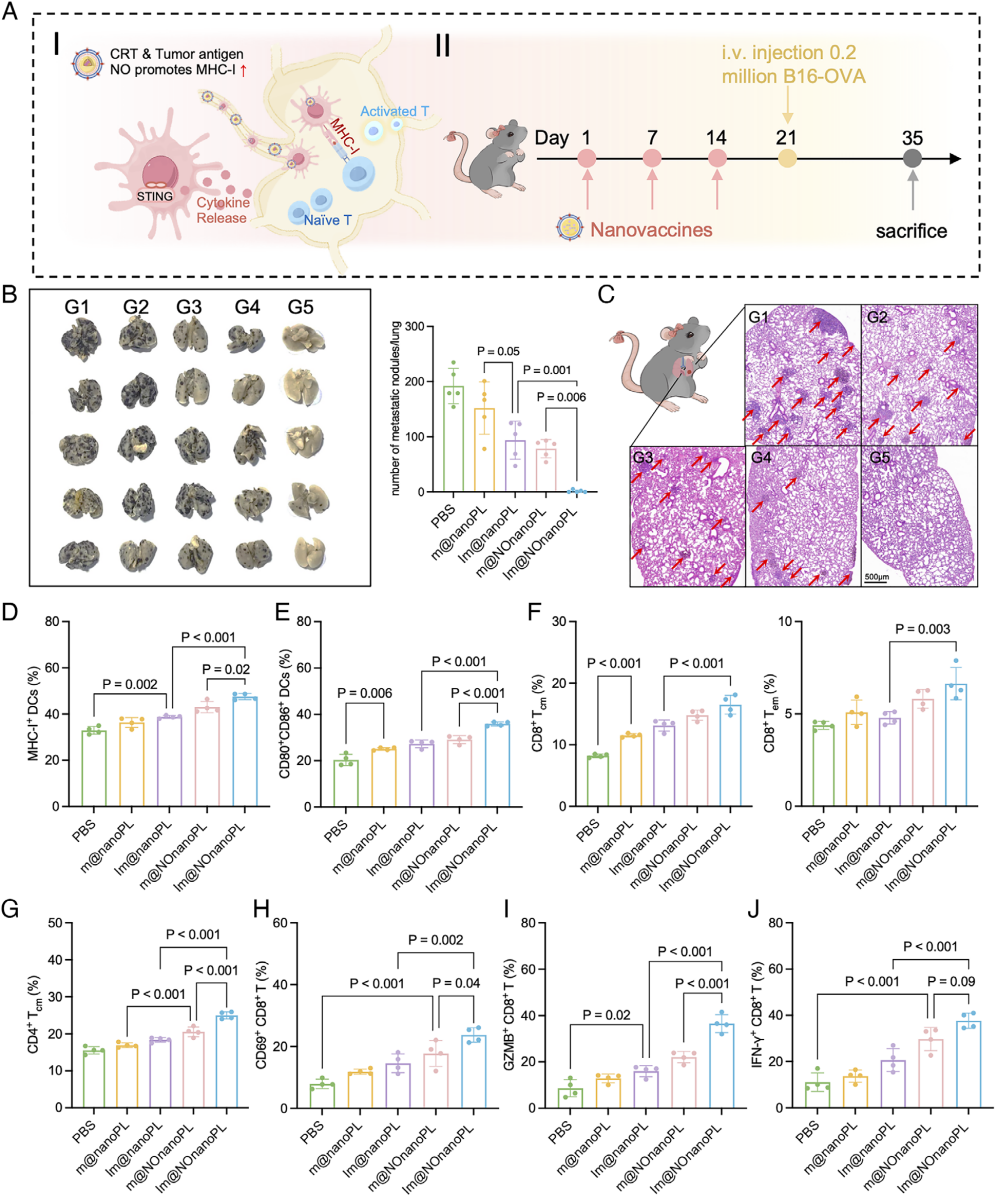
Prophylactic antitumor effects of nanovaccine in B16‐OVA cell pulmonary metastasis model. (A) Schematic representation of (I) The CRT‐coated tumor cell membrane on the surface of the Im@NOnanoPL nanovaccine delivers tumor antigens and targets lymph nodes; NO activates STING pathway and upregulates MHC‐I, enhancing CD8+ T cell immunity, and (II) mice receiving the indicated treatments. (G1: PBS, G2: m@nanoPL, G3: Im@nanoPL, G4: m@NOnanoPL, G5: Im@NOnanoPL) (B) Evaluation of lung metastasis in treated mice at day 35 post‐treatment by digital photograph and count tumor nodes. Statistical analysis was performed using one‐way ANOVA followed by Tukey's multiple comparisons test. (C) H&E staining images of lung metastasis, indicated by red arrows, scale bar 500 µm. (D) Flow cytometry analysis of MHC‐I expression on DCs in lymph nodes under different treatments (*n* = 4). (E) Flow cytometry analysis of CD80^+^ CD86^+^ among CD11c^+^ DCs in lymph nodes under different treatments (*n* = 4). (F and G) Percentages of the (F) CD8^+^ T_cm_, CD8^+^ T_em_ and (G) CD4^+^ T_cm_, in the spleens harvested from the mice at 35 days after first inoculation of different nanovaccine (*n* = 4). (H) Percentages of CD69^+^ CD8^+^ T cells in spleens detected by flow cytometry (*n* = 4). (I and J) Quantification of immune responses of CD8^+^ T cells in the spleen as measured by the percentage of Granzyme B^+^ or IFN‐γ^+^ among CD8^+^ T cells (*n* = 4). Statistical analysis was performed using the one‐way ANOVA with Tukey's multiple comparisons test.

The results revealed striking differences among treatment groups. While control formulations (m@nanoPL, Im@nanoPL, and m@NOnanoPL) exhibited limited antimetastatic efficacy, Im@NOnanoPL immunization resulted in a profound suppression of pulmonary metastasis, with virtually no detectable metastatic nodules in the lungs (Figure 6b). Histopathological analysis by H&E staining further corroborated these findings, showing preserved lung architecture and the absence of metastatic lesions in the Im@NOnanoPL‐treated group (Figure 6c). Together, these results demonstrate the superior antimetastatic efficacy of Im@NOnanoPL compared with control nanovaccine formulations.

To elucidate the underlying mechanisms, we next investigated how Im@NOnanoPL mediated its protective effect. Flow cytometry analysis of lymph node DCs demonstrated enhanced MHC‐I expression (Figure 6d) and elevated CD80^+^CD86^+^ populations (Figure 6e) in Im@NOnanoPL‐treated mice, indicating improved antigen presentation capacity. The vaccine also induced robust memory T cell responses, with significant increases in both CD8^+^ central memory (T_cm_, 2.1‐fold) and effector memory (T_em_, 1.8‐fold) populations compared to PBS controls (Figure 6f,g and Supplementary Figure S20). Subsequent antigen challenge experiments revealed heightened CD8^+^ T cell activation (CD69^+^CD8^+^T) and effector function, as evidenced by elevated IFN‐γ and GZMB production (Figure 6h–j). These findings collectively support a three‐phase protective mechanism: initial T cell priming, subsequent memory formation, and long‐term immune surveillance. The comprehensive immune activation induced by Im@NOnanoPL effectively prevented metastatic tumor establishment, highlighting its potential as a prophylactic cancer vaccine.

### 2.7 Therapeutic Efficacy of Im@NOnanoPL Nanovaccine Combined With αPD‐1 Checkpoint Inhibition

Building on the validated capability of Im@NOnanoPL to enhance immune surveillance and suppress tumor metastasis, we explored its potential as a therapeutic cancer vaccine to induce tumor regression and evaluated its synergistic effects with the clinically approved PD‐1 inhibitor (αPD‐1). In a subcutaneous MC38 colorectal cancer model (Figure 7a), after tumors grow to approximately 70 mm^3^, mice were randomized into six treatment groups: PBS control (G1), αPD‐1 monotherapy (G2), Im@nanoPL (G3), m@NOnanoPL (G4), Im@NOnanoPL (G5), and Im@NOnanoPL + αPD‐1 combination (G6). Subcutaneous vaccinations were administered on days 0 and 7, with αPD‐1 delivered intraperitoneally on day 4.

**FIGURE 7:**
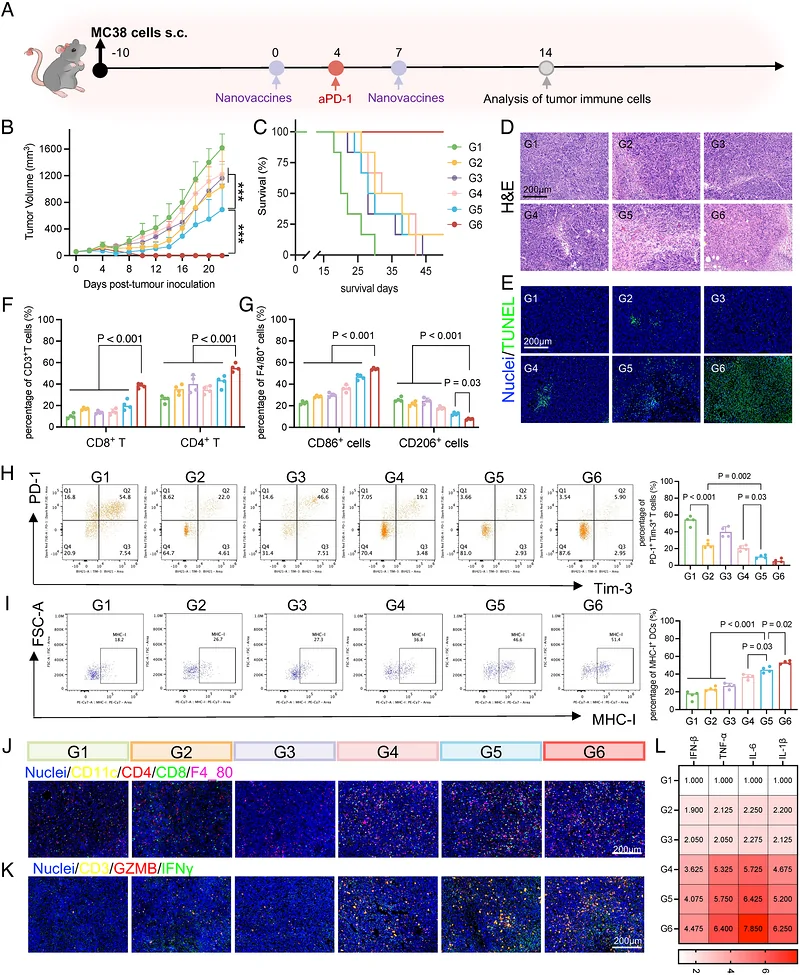
Therapeutic antitumor effects of Im@NOnanoPL nanovaccine on the MC38 colorectal cancer mouse model. (A) Schematic representation of mice receiving the indicated treatments. (B) Tumor growth profiles of the mice after different treatments. Tumor growth curves were analyzed using two‐way repeated‐measures ANOVA followed by Tukey's multiple comparisons test. (C) Survival curves of the mice after different treatments (*n* = 6 mice per group). (D) H&E staining images of MC38 tumor tissue, scale bar: 200 µm. (E) TUNEL staining (green) of tumors, Scale bar: 200 µm. (F) Flow cytometric quantification of the CD8^+^ or CD4^+^ T cells in the tumors (*n* = 4). (G) Flow cytometric quantification of the CD86^+^ F4/80^+^ or CD206^+^ F4/80^+^ cells in the tumors (*n* = 4). (H) Ratio of Exhaustion CD8^+^ T in the tumors characterized by flow cytometry (*n* = 4). (I) Flow cytometry analysis of MHC‐I expression on DCs in the tumors (*n* = 4). (J) Immunofluorescence staining for CD11c^+^ DCs (yellow), CD4^+^ T cell (red), CD8^+^ T cell (green), F4/80^+^ macrophages (pink) infiltration characterization. Scale bar: 200 µm. (K) Immunofluorescence staining for CD3^+^ T cell (yellow), GZMB (red), IFN‐γ (green). Scale bar: 200 µm. (L) Relative fold change of the pro‐inflammatory cytokines IFN‐β, TNF‐α, IL‐6, and IL‐1β in the TME. (G1: PBS, G2: αPD‐1, G3: Im@nanoPL, G4:m@NOnanoPL, G5: Im@NOnanoPL, G6: Im@NOnanoPL+ αPD‐1). Statistical analysis was performed using the one‐way ANOVA with Tukey's multiple comparisons test (*n* = 4).

Tumor growth analysis (Figure 7b) further highlighted the intrinsic therapeutic efficacy of Im@NOnanoPL and clarified the respective contributions of ICD membrane antigens and NO. Compared with PBS, Im@NOnanoPL monotherapy achieved a tumor growth inhibition rate of 57%, demonstrating that this nanovaccine alone is sufficient to exert a robust antitumor effect. By comparison, Im@nanoPL and m@NOnanoPL monotherapies resulted in only modest tumor inhibition (24% and 28%, respectively), indicating that ICD membrane antigens or NO alone provide limited therapeutic benefit. These results underscore that neither antigen delivery nor NO adjuvant activity alone is sufficient to elicit strong tumor control, whereas their integration within Im@NOnanoPL produces a markedly enhanced antitumor response. In contrast, αPD‐1 monotherapy resulted in only a ∼35% reduction in tumor volume, underscoring the limited efficacy of immune checkpoint blockade in this model when used alone. Importantly, the combination of Im@NOnanoPL with αPD‐1 markedly suppressed tumor growth, indicative of enhanced therapeutic efficacy with the combination treatment. This superior efficacy was durable and well tolerated, as no body weight loss was observed (Supplementary Figure S21). Consistently, all mice receiving the combination therapy remained tumor‐free with a 100% survival rate at day 50 (Figure 7c). Histopathological analyses corroborated these findings, revealing extensive tumor necrosis (Figure 7d), markedly reduced tumor cell proliferation (Supplementary Figure S22), and widespread apoptotic cell death (Figure 7e) in the Im@NOnanoPL + αPD‐1–treated group.

To elucidate the underlying mechanisms, tumors were harvested at 7 days post‐treatment for immune profiling. Flow cytometry revealed a 4.3‐fold increase in tumor‐infiltrating CD8^+^ T cells and a 2.1‐fold increase in CD4^+^ T cells in the Im@NOnanoPL + αPD‐1 group compared to PBS controls (Figure 7f, Supplementary Figure S23). Macrophage polarization shifted markedly toward the M1 phenotype (increasing from 22% to 53%) and away from the M2 phenotype (decreasing from 25.5% to 7.8%) (Figure 7g, Supplementary Figures S24–S25). NK cell infiltration (NK1.1^+^CD11b^+^) doubled relative to PBS (Supplementary Figure S26). Notably, reduced T cell exhaustion (PD‐1^+^Tim‐3^+^ T_ex_ populations) was observed not only in the αPD‐1 group but also in the m@NOnanoPL and Im@NOnanoPL monotherapy groups, with the most pronounced reduction in the combination group (from 55% to 5.9%) (Figure 7h). Additionally, mature DCs (CD80^+^CD86^+^CD11c^+^) and MHC‐I expression were significantly elevated in the combination group (Supplementary Figure S27 and Figure 7i).

Immunofluorescence staining (Figure 7j) confirmed enhanced infiltration of CD11c^+^ DCs, CD4^+^ T cells, CD8^+^ T cells, and F4/80^+^ macrophages in m@NOnanoPL, Im@NOnanoPL, and particularly the combination group. The Im@NOnanoPL + αPD‐1 group exhibited the highest IFN‐γ and GZMB production by CD3^+^ T cells, correlating with robust tumor‐killing activity (Figure 7k). ELISA quantification of tumor‐associated cytokines revealed dramatic increases in proinflammatory factors compared to PBS controls: IFN‐β (4.48‐fold), TNF‐α (6.4‐fold), IL‐6 (7.85‐fold), and IL‐1β (6.25‐fold) (Figure 7l). Collectively, these results demonstrate that Im@NOnanoPL combined with αPD‐1 achieves superior antitumor efficacy through three interconnected mechanisms: (1) potent activation of tumor‐specific T cell responses, (2) reversal of T cell exhaustion, and (3) remodeling of the immunosuppressive tumor microenvironment via proinflammatory cytokine amplification. This combinatorial strategy highlights the potential for clinical translation in enhancing cancer immunotherapy outcomes.

### 2.8 Long‐Term Immune Memory and Mechanisms of Im@NOnanoPL With αPD‐1 Combinatorial Therapy

To further elucidate the immunological mechanisms underlying the durable antitumor efficacy of Im@NOnanoPL combined with αPD‐1, we next characterized systemic immune responses and long‐term immune memory in MC38‐OVA tumor‐bearing mice according to the treatment regimen shown in Figure 8a. Flow cytometry of dLNs harvested at 7 days after final treatment showed that nanovaccine‐treated mice exhibited a 1.6‐fold increase in DC MHC‐I expression and a 1.9‐fold elevation in antigen‐MHC‐I complex formation compared to PBS controls (Figure 8b). The proportion of mature DCs (CD80^+^CD86^+^) increased from ∼24% in PBS controls to ∼52%, accompanied by significant expansion of CTLs: GZMB^+^ CD8^+^ T cells rose from 0.3% to 3.2% (*p* < 0.001), and IFN‐γ^+^ CD8^+^ T cells increased from 0.6% to 1.9% (*p* < 0.001) (Figure 8c,d). Parallel analysis of splenic T cells revealed enhanced activation (CD69^+^ populations) and effector function (elevated GZMB and IFN‐γ expression in both CD4^+^ and CD8^+^ T cells) following OVA stimulation (Figure 8e–g, Supplementary Figures S28 and S29). These findings collectively highlight systemic activation of the DC‐MHC‐I‐TCR axis, demonstrating the nanovaccine's capacity to amplify cross‐presentation efficiency and drive CTL clonal expansion.

**FIGURE 8:**
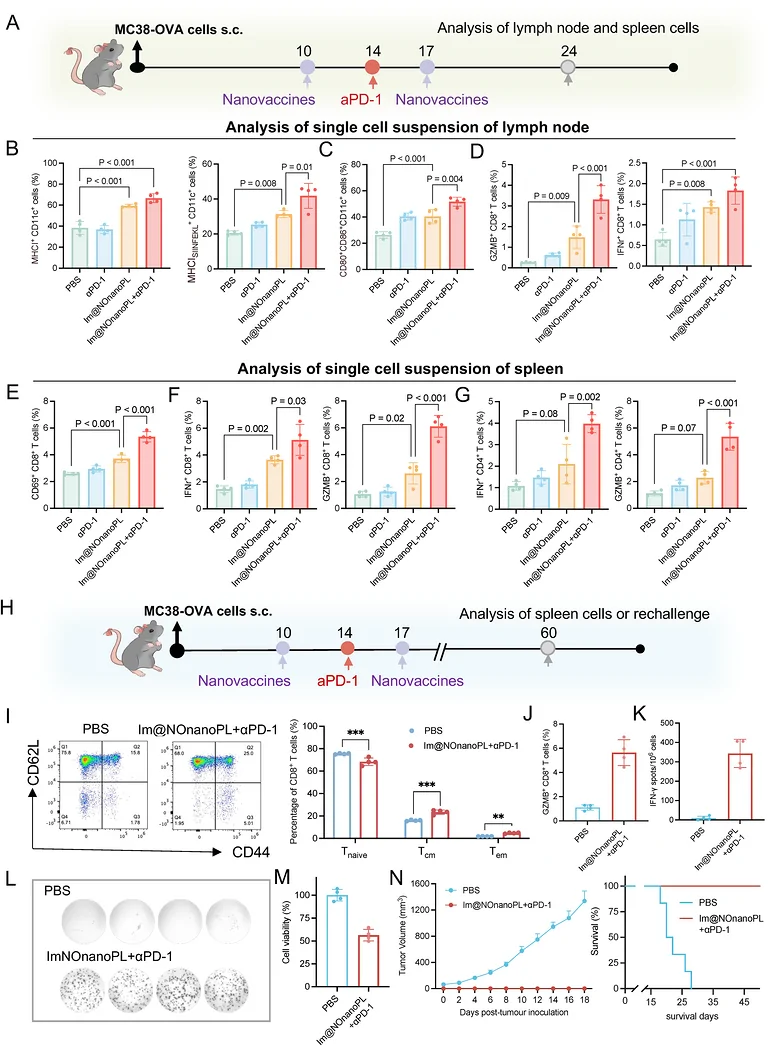
Im@NOnanoPL nanovaccine mediated enhanced long‐term immune memory in vivo. (A) Schematic illustration to evaluate the therapeutic antitumor effects after different treatments. (B) Flow cytometry analysis of MHC‐I expression and MHC‐I_OVA_ complex levels of DCs in lymph nodes (n = 4). Statistical analysis was performed using one‐way ANOVA followed by Tukey's multiple comparisons test. (C) Flow cytometry analysis of CD80^+^ CD86^+^ among CD11c^+^ DCs in lymph nodes (*n* = 4). Statistical analysis was performed using one‐way ANOVA followed by Tukey's multiple comparisons test. (D) Flow cytometry analysis of IFN‐γ and granzyme B expression on CD8^+^ T cells in lymph nodes (*n* = 4). Statistical analysis was performed using one‐way ANOVA followed by Tukey's multiple comparisons test. (E) Percentage of CD69^+^ CD8^+^ T cells in spleen after 24 h co‐culture with OVA polypeptide, as detected using flow cytometry (*n* = 4). Statistical analysis was performed using one‐way ANOVA followed by Tukey's multiple comparisons test. (F) Flow cytometry analysis of IFN‐γ and granzyme B expression on CD8^+^ T cells in spleen after 24 h co‐culture with OVA_257–264_ (*n* = 4). Statistical analysis was performed using one‐way ANOVA followed by Tukey's multiple comparisons test. (G) Flow cytometry analysis of IFN‐γ and granzyme B expression on CD4^+^ T cells in spleen after 24 h co‐culture with OVA polypeptide. Statistical analysis was performed using one‐way ANOVA followed by Tukey's multiple comparisons test. (H) Schema illustrating the vaccination long‐term immune memory. Mice were challenged with MC38‐OVA cells (3 × 10^5^ cells/mouse, s.c.) on day 0. Mice with complete tumor regression were subjected to tumor rechallenge with MC38‐OVA cells (3 × 10^6^ cells/mouse, s.c.), and tumor analysis was performed on day 60. (I) Representative flow cytometry images of central T cells and memory T cells in spleen following various treatments. Statistical analysis was performed using one‐way ANOVA followed by Tukey's multiple comparisons test. (J) Flow cytometry analysis of granzyme B expression on CD8^+^ T cells in spleen after 24 h co‐culture with OVA_257–264_. (K and L) IFN‐𝛾 secretion by splenocytes isolated from different treatment groups, as determined using the ELISPOT assay after restimulation of the cells with OVA_257–264_. (M) Tumor killing ability of the splenocytes isolated T cells activated by OVA_257–264_. After 24 h co‐culture with OVA_257–264_, the T cells were co‐cultured with MC38‐OVA cells at a 3:1 ratio for 24 h, cell viability was measured by CCK‐8 assay. (N) Tumor growth curve following tumor rechallenge and Survival curves of mice under Im@NOnanoPL+αPD‐1 or PBS. (*n* = 6).

To evaluate the critical challenge of long‐term tumor control, we investigated the durability of immune memory responses. At day 60 after tumor inoculation, cured mice were divided into two cohorts: one for splenic memory T cell analysis and the other for contralateral tumor rechallenge alongside age‐matched naive controls (Figure 8h). The Im@NOnanoPL + αPD‐1 group exhibited significantly enriched memory T cell populations compared with controls, with CD8^+^ T_cm_ (central memory) increasing from 15.8% to 25% (*p* < 0.001) and CD8^+^ T_em_ (effector memory) rising from 1.78% to 5.01% (*p* < 0.01) (Figure 8i). OVA restimulation further enhanced GZMB expression in both CD4^+^ and CD8^+^ T cells, with GZMB^+^ CD8^+^ T cells showing a 4‐fold increase over PBS controls (Figure 8j). ELISPOT analysis confirmed superior IFN‐γ production in splenocytes from combination‐treated mice (Figure 8k,l), while co‐culture experiments demonstrated potent tumoricidal activity, with ∼49% specific cytotoxicity against MC38 cells (Figure 8m). Consistent with these memory responses, tumor rechallenge experiments revealed complete protection in Im@NOnanoPL + αPD‐1‐treated mice, with no tumor recurrence observed during the monitoring period and 100% survival maintained (Figure 8n). These data demonstrate that the combinatorial strategy establishes durable, tumor‐specific immune memory capable of preventing relapse and mediating long‐term antitumor surveillance.

### 2.9 Biosafety Evaluation

To further assess the in vivo biosafety of Im@NOnanoPL, we evaluated its blood compatibility, hematological parameters, and potential long‐term tissue toxicity. As described above, in vivo fluorescence imaging indicated that Im@NOnanoPL was predominantly cleared via the liver and kidneys, suggesting normal metabolic elimination pathways. Systemic blood compatibility was examined using a hemolysis assay (Supplementary Figure S30). No detectable hemolysis was observed even at an Im@NOnanoPL concentration of 200 µg mL^−^^1^, indicating minimal acute blood toxicity. In addition, routine blood analysis performed at 1 and 7 days after administration showed no significant alterations in hematological parameters compared with control mice (Supplementary Figure S31). To evaluate potential long‐term tissue toxicity, major organs, including the heart, liver, spleen, lung, and kidney, were collected 18 days after treatment and subjected to histopathological examination. H&E staining revealed no observable pathological abnormalities or inflammatory damage in any of the examined organs (Supplementary Figure S32). Collectively, these results demonstrate that Im@NOnanoPL exhibits favorable in vivo biosafety under the tested therapeutic regimen.

## 3 Conclusions

The Im@NOnanoPL nanovaccine platform exemplifies a rationally engineered strategy to synergize nitric oxide‐driven immunomodulation with antigen delivery via ICD membranes, establishing a coordinated system for robust antitumor immunity. Central to its efficacy is the novel NO‐enhanced POLY(I:C) adjuvant system: (1) activation of the STING‐TBK1‐IRF3 signaling axis, as evidenced by phosphorylation‐dependent upregulation of downstream effectors (p‐TBK1, p‐STING), and (2) enhancement of MHC‐I‐mediated antigen presentation in DCs and macrophages. Complementing this, ICD membranes derived from cisplatin‐treated tumor cells provide a comprehensive endogenous antigen repertoire, enriched with DAMPs, to promote antigen uptake and lymph node targeting, while circumventing artificial neoantigen selection bias. A concise comparison highlighting the superior immunopotency, favorable safety profile, and efficient lymphoid delivery of Im@NOnanoPL relative to conventional STING agonists is provided in Table S1.

In prophylactic models, Im@NOnanoPL demonstrated near‐complete protection against B16‐OVA lung metastasis. When combined with αPD‐1 checkpoint blockade, the platform achieved synergistic therapeutic outcomes, eradicating established MC38‐OVA tumors and establishing durable immune memory. In summary, Im@NOnanoPL integrates NO‐driven immunomodulation with ICD‐derived antigens, and its NO‐enhanced POLY(I:C) adjuvant robustly promotes CD8^+^ T cell responses. This facile platform may extend beyond POLY(I:C), offering broad applicability and strong translational potential for cancer immunotherapy.

## 4 Methods

### 4.1 Materials and Reagents

NOC‐18 and DOTAP were sourced from MedChemExpress (MCE, China), while POLY(I:C) was obtained from Invivogen (USA). PLGA (lactide: glycolide = 50:50) and DSPE‐PEG_2000_ were custom‐synthesized by RuiXi Biological Technology Co., Ltd. (China). The OVA_257‐264_ peptide (SIINFEKL) was procured from Anhui Guoping Pharmaceutical Co., Ltd. (China). Cell culture reagents, including RPMI‐1640 medium and fetal bovine serum, were purchased from Vivacell (Beijing Lablead Science & Technology Co., Ltd., China). Mouse ELISA kits for quantifying IL‐6, IL‐1β, TNF‐α, and IFN‐β were supplied by Beijing Solarbio Science & Technology Co., Ltd. (China). DAPI, PMSF, and RIPA lysis buffer were acquired from Beyotime Biotechnology (China). Antibodies targeting phospho‐TBK1 (Ser172, #5483), phospho‐STING (Ser365, #72971), total TBK1 (#38066), and total STING (#13647) were purchased from Cell Signaling Technology (CST) via Beijing Chengzhi Kewei Biotechnology Co., Ltd. (China). All reagents were stored and used according to manufacturer specifications to ensure experimental reproducibility.

For Flow Cytometry Antibody: PE/Cyanine7 anti‐mouse CD45 Antibody (BioLegend, 30‐F11, 103114); PE Dazzle 594 anti‐mouse NK1.1 Antibody (BioLegend, PK136, 108747)**;** PE anti‐mouse H‐2K_b_ SIINFEKL Antibody (BioLegend, 25‐D1.16, 141603)**;** PE/Cyanine7 anti‐mouse H‐2K_d_ Antibody (BioLegend, SF1‐1.1, 116622)**;**APC anti‐mouse CD25 Antibody (BioLegend, PC61, 102012)**;** APC anti‐mouse CD11c Antibody (BioLegend, N418, 117310)**;** Spark Red 718 anti‐mouse PD‐1 (CD279) Antibody (BioLegend, 29F.1A12, 135259)**;** FITC anti‐human CD8a Antibody (BioLegend, RPA‐T8, 301060)**;** Brilliant Violet 421 anti‐mouse Tim‐3 (CD366) Antibody (BioLegend, A18087E, 364808)**;** FITC anti‐mouse CD11b Antibody (BioLegend, M1/70, 101205)**;** APC anti‐mouse F4/80 Antibody (BioLegend, BM8, 123116)**;**Brilliant Violet 785 anti‐mouse CD86 Antibody (BioLegend, GL‐1, 105043)**;** PE/Cyanine5 anti‐mouse CD206 (MMR) Antibody (BioLegend, C068C2, 141739)**;** Brilliant Violet 711 anti‐mouse CD80 Antibody (BioLegend, 16‐10A1, 104743)**;** Brilliant Violet 650 anti‐mouse CD11c Antibody (BioLegend, N418, 117339)**;** Brilliant Violet 421 anti‐mouse Tim‐3 (CD366) Antibody (BioLegend, B8.2C12, 134019);BUV395 Anti‐Mouse CD3 Antibody (Invitrogen, 17A2, 363‐0032‐82); BUV563 Anti‐Mouse CD4 Antibody (Invitrogen, GK1.5, 365‐0041‐82); True‐Stain Multi‐Fluor Buffer (BioLegend, 426105); TruStain FcX PLUS (anti‐CD16/32) Antibody (BioLegend, 156603).

### 4.2 Animals and Cells

Female C57BL/6 mice (6 weeks old) were obtained from the Department of Laboratory Animal Science at Peking University Health Science Center. All experimental procedures were approved by the Institutional Animal Care and Use Committee of Peking University Third Hospital. Animal husbandry and experimental protocols were prospectively reviewed and authorized by the ethics committee prior to study initiation. MC38, MC38‐OVA, B16, and B16‐OVA cell lines were maintained in high‐glucose Dulbecco's modified Eagle's medium (DMEM; VivaCell) supplemented with 10% fetal bovine serum (FBS; VivaCell) and 1% penicillin‐streptomycin (VivaCell), under standard culture conditions (37°C, 5% CO_2_, humidified atmosphere). The DC2.4 cell line used in this study was generously provided by the research group of Prof. Gang Liu at Xiamen University. CD3^+^ T cells were isolated from spleens of MC38 tumor‐bearing mice using the MojoSort Mouse CD3 T Cell Isolation Kit (BioLegend) through negative magnetic sorting.

### 4.3 Synthesis of Im@NOnanoPL

The NOnanoPL formulation was synthesized through a sequential nanoprecipitation and self‐assembly process. Stock solutions were prepared as follows: POLY(I:C) in ddH_2_O (4 mg mL^−^^1^), DOTAP in ethanol (2 mg mL^−^^1^), PLGA in DMSO (5 mg mL^−^^1^), NOC‐18 in ddH_2_O (25 mg mL^−^^1^), and DSPE‐PEG_2000_ in ddH_2_O (1 mg mL^−^^1^). First, 100 µL of POLY(I:C) (4 mg mL^−^^1^) was mixed with 10 µL of NOC‐18 (25 mg mL^−^^1^) under vortex agitation. This mixture was then injected dropwise into 100 µL of DOTAP (2 mg mL^−^^1^) under continuous sonication in a water bath (40 kHz, 400 W) for 1 min, forming a primary nucleic acid–lipid complex stabilized mainly by electrostatic interactions between the negatively charged POLY(I:C) and the positively charged DOTAP. The resulting primary complex was subsequently added to 200 µL of PLGA (5 mg mL^−^^1^) under sustained sonication for an additional 1 min to form a polymeric core‐shell structure, in which hydrophobic interactions between the lipid tails and PLGA help stabilize the encapsulated adjuvant and NO donor. Finally, the PLGA composite was transferred into 1200 µL of DSPE‐PEG_2000_ (1 mg mL^−^^1^) and sonicated for 1 min to achieve PEGylated nanoparticle stabilization; here, hydrophobic interactions between the DSPE lipid tails and PLGA stabilize the particles in aqueous solution, while PEG chains enhance colloidal stability. For nanoPL preparation, water was used instead of the NOC‐18 component, and the other preparation methods were the same as for NOnanoPL. DSPE‐PEG modification was introduced to improve the aqueous dispersibility and colloidal stability of PLGA nanoparticles during membrane coating in water or PBS, thereby preventing aggregation and facilitating uniform ICD cell membrane cloaking.

Im‐Vesicles were derived from MC38‐OVA cells cultured in 10 cm dishes. Upon reaching 80% confluence, cells were treated with 80 µM cisplatin for 24 h to induce immunogenic cell death (ICD). ICD status was confirmed via flow cytometry and confocal microscopy analysis of hallmark markers. Cells were then harvested using a cell scraper into ice‐cold PBS containing 1 mM PMSF (protease inhibitor) and subjected to ultrasonic disruption (1 W cm^−^^2^, 10 s total duration with 2 s pulses followed by 3 s intervals) on ice. Cellular debris and organelles were removed through sequential differential centrifugation. The resulting membrane pellet was resuspended in PBS and extruded through a 400 nm polycarbonate membrane using a mini‐extruder (Avanti Polar Lipids) to form uniform Im‐Vesicles. Control m‐Vesicles were prepared identically from untreated MC38‐OVA cells without cisplatin exposure. Vesicle size and protein content were quantified by dynamic light scattering (Malvern Zetasizer) and BCA assay (Thermo Fisher); ICD status was confirmed via flow cytometry. Final preparations are stored at −80°C until use.

The synthesis of Im@NOnanoPL was achieved through a cell membrane‐coating strategy. Purified Im‐vesicles (derived from cisplatin‐induced immunogenic cell death of MC38‐OVA cells) were mixed with pre‐synthesized NOnanoPL nanoparticles at a mass ratio of 3:1 (membrane protein: PLGA core) in PBS buffer. The mixture was homogenized via bath sonication for 3 min and subsequently extruded through a liposome extruder (Avanti Polar Lipids) equipped with a 200 nm polycarbonate membrane for 11 cycles to enable directional membrane coating of the nanoparticles. The final product was resuspended in sterile PBS for further use. Using identical protocols, control formulations were prepared: m@NOnanoPL (using membrane vesicles from untreated cells), Im@nanoPL (lacking NOC‐18), and m@nanoPL (using untreated cell membranes and lacking NOC‐18). Physicochemical characterization included dynamic light scattering (DLS) for hydrodynamic diameter and polydispersity index (PDI), zeta potential analysis for surface charge, and transmission electron microscopy (TEM) for morphological visualization.

### 4.4 Isolation and Culture of BMDCs

BMDCs were generated from C57BL/6 mice (6–8 weeks old) as previously described with modifications. Briefly, femurs and tibias were aseptically removed after euthanasia and cleaned of surrounding muscle tissue. Bone marrow was flushed out using a 25‐gauge needle with cold RPMI‐1640 medium, and the cell suspension was filtered through a 70‐µm cell strainer. After centrifugation at 300 × g for 5 min, red blood cells were lysed with Red Cell lysis Solution (solarbio) for 3 min at room temperature. The remaining cells were resuspended in complete RPMI‐1640 medium supplemented with 10% FBS, 20 ng mL^−1^ recombinant mouse GM‐CSF, and 10 ng mL^−1^ IL‐4. Cells were plated at a density of 3×10^6^ cells mL^−1^ in 6‐well plates and maintained at 37°C in a 5% CO_2_ humidified incubator. On day 3, half of the medium was carefully replaced with fresh cytokine‐containing medium. After 5 days of culture, non‐adherent and loosely adherent cells were collected and characterized by flow cytometry for CD11c^+^MHC‐II^+^CD80^+^CD86^+^ expression.

Single‐cell suspensions were prepared from spleens of C57BL/6 mice by mechanical dissociation through a 70‐µm cell strainer in RPMI‐1640 medium. After red blood cells were lysed with Red Cell lysis Solution (Solarbio) for 3 min at room temperature, splenocytes were washed and resuspended in PBS without Ca/Mg. CD3^+^ T cells were isolated using the MojoSort Mouse CD3 T Cell Isolation Kit (cat: 480024) according to the manufacturer's protocol. CD3^+^T is characterized by flow cytometry for CD45^+^CD3^+^ expression.

### 4.5 Cytokines Detection

To evaluate the immunomodulatory effects of nanovaccine formulations on DC‐T cell crosstalk, BMDCs were first incubated with different nanovaccine groups (100 µM NOC‐18) for 24 h, followed by co‐culture with purified CD8^+^ T cells at a 1:10 ratio (BMDC: T cells) for an additional 24 h in complete RPMI‐1640 medium. Cell culture supernatants were then collected and analyzed for secreted cytokines (IL‐1β, TNF‐α, IL‐6, and IFN‐β) using BioLegend ELISA MAX Deluxe kits according to the manufacturer's protocols. All measurements were performed in technical triplicates from four independent biological replicates, with standard curves generated using recombinant cytokines for quantitative analysis. The BMDC and CD8^+^ T cell isolation procedures followed established protocols as previously described in the Supplementary Material. This co‐culture system enables comprehensive assessment of nanovaccine‐induced dendritic cell maturation and subsequent T cell activation through quantitative profiling of key immunomodulatory cytokines in the supernatant.

### 4.6 Quantitative Analysis of Cellular Internalization and Lysosomal Trafficking

To quantify the time‐dependent uptake efficiency of the Cy5‐labeled nanovaccine by DC2.4 cells (murine dendritic cell line), cells were seeded at 2 × 10^5^ cells/well in 12‐well plates and allowed to adhere for 12 h. Cy5‐labeled NPs (Cy5 concentration 1 µg mL^−^^1^) were added to the culture medium (RPMI‐1640 + 10% FBS) and incubated at 37°C under 5% CO_2_ for 1, 2, 3, 4, and 6 h. At each time point, cells were washed three times with PBS to remove the nanovaccines that were not taken up by the cells. Cells were digested with trypsin and then washed off with PBS and resuspended in PBS. Flow cytometry was performed using a BD FACSCelesta system with the following settings: Cy5 channel (640 nm excitation, 660/20 nm emission), thresholding on FSC/SSC to exclude debris. For each sample, 10,000 live cell events were acquired. Data were analyzed using FlowJo v10.8, with fluorescence intensity normalized to the 0 h control.

DC2.4 cells were seeded in confocal dishes (5 × 10^4^ cells/well) and incubated with Cy5‐labeled nanovaccine (50 µg mL^−^^1^) for 2 h or 6 h, followed by 50 nM LysoTracker Green (30 min, 37°C) to label lysosomes. Imaging was performed on a Nikon A1R HD25 confocal microscope using a 60× oil objective. LysoTracker Green was excited at 488 nm (500‐550 nm emission), and Cy5 at 640 nm (660‐720 nm emission). Results showed time‐dependent changes in nanovaccine‐lysosome association, with significant lysosomal escape by 6 h.

### 4.7 IFN‐𝛾 ELISPOT Assays

In the MC38‐OVA subcutaneous tumor model, vaccinations with Im@NOnanoPL (containing 0.25 mg mL^−^^1^ POLY(I:C)) were administered subcutaneously on days 10 and 17 post‐tumor implantation, followed by intraperitoneal injection of αPD‐1 (100 µg/mouse) on day 14. Sixty days after tumor inoculation, spleens were harvested from treated mice and age‐matched healthy controls for immune memory evaluation. Splenic single‐cell suspensions were prepared by mechanical dissociation through a 70‐µm cell strainer and washed twice with PBS prior to resuspension in RPMI‐1640 medium supplemented with 10% FBS.

The IFN‐γ ELISPOT assay was performed using the Mouse IFN‐γ ELISPOT Kit (Dakewe) according to the manufacturer's protocol. Briefly, ELISPOT plates were prehydrated with RPMI‐1640 medium for 10 min at 37°C, followed by seeding of freshly isolated splenocytes (2 × 10^5^ cells/well in 100 µL medium). Cells were stimulated with OVA peptide (10 µg mL^−^^1^ final concentration). After 24 h of incubation at 37°C under 5% CO_2_, cells were removed, and residual cells were lysed by incubation with pre‐cooled ddH_2_O for 10 min at 4°C. Removed ddH_2_O, plates were subsequently incubated with biotinylated detection antibody for 2 h at room temperature, washed six times, and treated with streptavidin‐HRP conjugate for 1 h. Following additional washes, spots were developed using AEC substrate solution, with reactions terminated by rinsing with distilled water. Air‐dried plates were analyzed using an automated ELISPOT reader (Cellular Technology Ltd), and spot counts were quantified with ImmunoSpot software.

### 4.8 Determination of Lymphatic Targeting

Cy5‐Im@NOnanoPL was synthesized by conjugating hydrophilic Cy5‐NHS ester (300 µg mL^−^^1^) with Im‐vesicles (3 mg mL^−^^1^ membrane protein) in PBS at 4°C for 12 h. Unbound Cy5‐NHS was removed via centrifugation at 10,000 × g, followed by three PBS washes. The Cy5‐labeled Im‐vesicles were subsequently encapsulated onto NOnanoPL at a mass ratio of 3:1 (vesicles:core) through bath sonication for 3 min, followed by extrusion through a lipid extruder to form Cy5‐Im@NOnanoPL nanoparticles. Mice received subcutaneous injections of either Cy5‐Im@NOnanoPL or Cy5‐m@NOnanoPL proximal to the right hind limb. At 12, 24, and 36 h post‐injection, mice were euthanized, and draining lymph nodes were excised for ex vivo fluorescence imaging using an IVIS spectrum imaging system. Fluorescence intensity was quantified using Living Image software.

### 4.9 Analysis of the Immune Response In Vivo

Six‐week‐old C57BL/6N mice were subcutaneously immunized in the right flank with 100 µL of formulated vaccines (Group I: PBS, II: m@nanoPL, III: Im@nanoPL, IV: m@NOnanoPL, V: Im@NOnanoPL) containing 250 µg mL^−^^1^ POLY(I:C) on days 0 and 7. At day 14 post‐immunization, bilateral draining lymph nodes (dLNs) and spleens were harvested for immune profiling. Lymph nodes were mechanically dissociated through a 70‐µm cell strainer, while splenic single‐cell suspensions were prepared by sequential mechanical disruption and red blood cell lysis, with all procedures performed on ice to preserve cell viability.

For flow cytometry analysis, lymph node‐derived cells were stained with the following BioLegend antibodies: CD11c‐BV510, CD4‐FITC, CD3‐PE‐Cy5, CD8‐PerCP‐Cy5.5, MHC‐I‐PE‐Cy7, MHC‐I‐OVA‐APC‐Fire750, CD80‐BV711, CD86‐BV785, GZMB‐PE, and IFN‐γ‐BV421. Splenocytes were resuspended in RPMI‐1640 medium and cultured with OVA peptide (10 µg mL^−^^1^) for 24 h. Supernatants were collected for ELISA quantification of IFN‐β, IL‐1β, IL‐6, and TNF‐α using commercial kits (R&D Systems).

For splenocytes, Brefeldin A (1× GolgiPlug, BioLegend) was added for the final 4 h of incubation to inhibit cytokine secretion. Cells were incubated with Fc Receptor Blocking Solution (anti‐CD16/32 antibody, BioLegend) diluted in Cell Staining Buffer (1:100) for 15 min at 4°C. Cells were washed with PBS and stained with surface markers (CD3‐PE‐Cy5, CD4‐FITC, and CD8‐PerCP‐Cy5.5) in Cell Staining Buffer (BioLegend) for 20 min at 4°C. Following staining with surface antibodies, cells were fixed in fixation buffer for 15 min at 4°C and washed with PBS. Subsequent permeabilization was performed using Intracellular Staining Permeabilization Wash Buffer (10X, BioLegend), diluted to 1× working concentration according to the manufacturer's instructions, to ensure optimal membrane disruption while maintaining cellular morphology. Cells were then incubated with anti‐IFN‐γ‐BV421 and anti‐GZMB‐PE antibodies diluted in permeabilization buffer for 20 min on ice under light‐protected conditions to minimize fluorophore degradation. After staining, cells were washed twice with 1× permeabilization buffer to remove unbound antibodies, followed by a final wash in cell staining buffer (BioLegend) to quench residual permeabilization agents. Processed cells were resuspended in fresh cell staining buffer and stored at 4°C in the dark for immediate acquisition or short‐term preservation prior to flow cytometric analysis. Antibody dilution concentrations were as per the manufacturer's instructions.

### 4.10 Evaluation of Anti‐Metastasis Effect

Vaccinations were administered subcutaneously in the right hind flank on days 1, 7, and 14 to six‐week‐old C57BL/6N mice divided into five groups (G1: PBS, G2: m@nanoPL, G3: Im@nanoPL, G4: m@NOnanoPL, G5: Im@NOnanoPL). To establish the lung metastasis model, 0.2 × 10^6^ B16‐OVA tumor cells were intravenously injected via the tail vein into each mouse 7 days after the final vaccination. Mice were sacrificed on day 35 post‐tumor inoculation for comprehensive analysis. Lung tissues were harvested to quantify metastatic foci, and histological evaluation was performed using H&E staining. Concurrently, draining lymph nodes and spleens were collected for immune cell profiling.

### 4.11 In Vivo Anti‐Tumor Efficacy

The subcutaneous tumor model was established by inoculating 3 × 10^5^ MC38‐OVA cells into the right flank of six‐week‐old C57BL/6N mice. Tumor growth was monitored until day 10 post‐implantation, at which point tumors reached an average volume of approximately 70 mm^3^. Mice with comparable tumor sizes were evenly randomized into six groups: G1 (PBS control), G2 (αPD‐1 monotherapy), G3 (Im@nanoPL), G4 (m@NOnanoPL), G5 (Im@NOnanoPL), and G6 (Im@NOnanoPL + αPD‐1). Groups G3–G6 received subcutaneous vaccinations in the right flank (250 µg mL^−^^1^ POLY(I:C), 100 µL per mouse) on days 0 and 7 post‐tumor implantation, while G2 and G6 were administered αPD‐1 (100 µg per mouse) intraperitoneally on day 4. Seven days after the final vaccination (day 24 post‐tumor implantation), a subset of mice (*n* = 4/group) were sacrificed for immune profiling. Tumor tissues were analyzed by immunofluorescence staining to evaluate immune cell infiltration, and single‐cell suspensions were prepared for flow cytometric characterization of tumor‐infiltrating lymphocytes (TILs).

Flow cytometric analysis was performed using antibodies from BioLegend and Thermofisher to characterize immune cell populations and functional markers. Cell viability was assessed with Zombie Yellow Fixable Viability Kit (1:1000 dilution), followed by Fc receptor blocking with anti‐CD16/32 (clone 93) prior to surface marker staining. The following antibody panel was employed in a 16‐color configuration: CD45‐PE‐Cy7, NK1.1‐PE Dazzle 594, CD11b‐FITC, F4/80‐APC, CD86‐BV785, CD206‐PE‐Cy5, CD11c‐BV650, CD80‐BV711, MHC‐I‐PE‐Cy7, Tim‐3‐BV421, PD‐1‐Spark Red 718, CD3‐BUV395, CD8‐FITC, and CD4‐BUV563. Cells were incubated with antibody cocktails for 20 min at 4°C in Cell Staining Buffer (BioLegend), protected from light. Following surface staining, cells were fixed with fixation buffer for 15 min at room temperature. Samples were washed with PBS and resuspended in Cell Staining Buffer acquired on a Sony ID700 spectral flow cytometer, with data analyzed using FlowJo v10.8 software (BD Biosciences).

Remaining mice (*n* = 6/group) continued to be monitored for tumor volume (measured by digital caliper) and body weight changes until study endpoints. Tumor volumes were calculated using the formula: Volume = (Length × Width^2^)/2.

### 4.12 Rechallenge Study

In the MC38‐OVA tumor rechallenge model, 3 × 10^5^ MC38‐OVA cells were subcutaneously implanted into the right flank of C57BL/6N mice (*n* = 6/group). When tumors reached ∼70 mm^3^ (day 10 post‐implantation), the mice received subcutaneous vaccination on days 10 and 17 post‐tumor implantation, followed by intraperitoneal administration of αPD‐1 on day 14. After complete tumor eradication, cured mice underwent contralateral rechallenge with 3 × 10^6^ MC38‐OVA cells (10× primary tumor inoculum) in the left flank, while age‐matched naive mice served as controls. Tumor growth was monitored every two days using digital calipers, with volumes calculated as (length × width^2^/2). Survival was tracked until day 60 post‐rechallenge or until humane endpoints (tumor volume >1500 mm^3^). For immune memory analysis, spleens were harvested to quantify antigen‐specific T cell responses.

### 4.13 Statistical Analysis

Experimental data are presented as mean ± SD unless otherwise specified. The exact sample size (n) and statistical method for each experiment are indicated in the corresponding figure legends. All statistical analyses were performed using GraphPad Prism software (version 10.3.1; GraphPad Software Inc.). Specific statistical methods (e.g., two‐tailed unpaired Student's *t*‐test, one‐way ANOVA with Tukey's post hoc test) for individual experiments are explicitly described in figure captions. Statistical significance thresholds were defined as follows: *p* > ≥ 0.05 (not significant, ns), *p* < 0.05 (*), *p* < 0.01 (**), and *p* < 0.001 (***).

## Author Contributions

**Shuyu Xu**: conceptualization, methodology, performed the experiments, writing – original draft, writing – review and editing. **Yang Song**: performed the experiments, writing – review and editing. **Tianjiao Zhang**: conceptualization, methodology, performed the experiments, writing – review and editing. **Yan Wang**: Performed the experiments. **Mengxin Wang**: performed the experiments. **Xinxin Xie**: performed the experiments. **Qingfeng Chen**: methodology. **Xiaolong Liang**: conceptualization, supervision, writing – review and editing

## Ethics Statement

All experimental procedures were approved by the Institutional Animal Care and Use Committee of Peking University, assigned numbers DLASBE0313.

## Conflicts of Interest

The authors declare no conflicts of interest.

## Supporting information




**Supporting File**: exp270238‐sup‐0001‐SuppMat.docx.

## Data Availability

All data are available in the main text or the supplementary materials.
