# Supplementary material for: A Nitric Oxide–Based STING Agonist Nanoadjuvant With ICD‐Derived Membrane Antigens Unleashes Robust Antitumor Immunity
Source: Exploration (Beijing). 2026 Oct 11;6(5):70238. doi: 10.1002/exp2.70238 (PMC13654190; doi:10.1002/exp2.70238)
Supplement: Supplementary file 1 [file EXP2-6-70238-s001.docx]

Supplementary Materials for

**A Nitric Oxide–Based STING Agonist Nanoadjuvant with ICD-Derived Membrane Antigens Unleashes Robust Antitumor Immunity**

Shuyu Xu,^1†^ Yang Song,^1,2†^ Tianjiao Zhang,^1†^ Yan Wang,^3^ Mengxin Wang,^1^ Xinxin Xie,^1^ Qingfeng Chen,^4^ Xiaolong Liang,^1,5*^

^1^ Beijing Key Laboratory of Ultrasound Targeted Drug Delivery and Frontier Imaging Technology, Department of Ultrasound, Peking University Third Hospital, Beijing 100191, China.

^2^ Departments of Ultrasound Medicine Beijing Chaoyang Hospital, Capital Medical University, North Road 8, Gongti, Chaoyang, Beijing 100020, China

^3^ Department of Ultrasound, The First Affiliated Hospital of China Medical University, Shenyang 110001, China

^4^ Institute of Molecular and Cell Biology, Agency for Science, Technology and Research, Singapore 138673, Singapore

^5^ State Key Laboratory of Vascular Homeostasis and Remodeling, Beijing Key Laboratory of Interdisciplinary Research in Gastrointestinal Oncology (BLGO), Peking University Third Hospital, Beijing 100191, China

^†^ These authors contributed equally

^*^ Correspondence: xiaolong_liang@bjmu.edu.cn

**Figure S1.** Flow cytometry analysis of MHC-I levels in BMDCs after 24h incubation with NOC-18 (0, 20, 50, 100, 150, 200 μM), as detected by flow cytometry (n=4). The data were shown as mean ± SD. Statistical analysis was performed by One-way ANOVA with a Tukey multiple comparisons test. Other groups were compared with the 0 μM group. *P<0.05; **P< 0.01; ***P< 0.001.

**Figure S2.** Flow cytometry analysis of MHC-I levels in BMDMs after 0, 12, 24, and 36 h incubation with NOC-18 (150 μM), as detected by flow cytometry (n=4). The data were shown as mean ± SD. Statistical analysis was performed by One-way ANOVA with a Tukey multiple comparisons test.

**Figure S3.** Flow cytometry analysis of CD206 levels in BMDMs after 0, 12, 24, and 36 h incubation with NOC-18, as detected by flow cytometry (n=4). The data were shown as mean ± SD. Statistical analysis was performed by One-way ANOVA with a Tukey multiple comparisons test.

**Figure S4.** Flow cytometry analysis of MHC-I_OVA_ complex levels in BMDCs after 0, 12, 24, and 36 h incubation with GSNO (800 μM), as detected by flow cytometry (n=4). The data were shown as mean ± SD. Statistical analysis was performed by One-way ANOVA with a Tukey multiple comparisons test. Other groups were compared with the 0 h group. *P<0.05; **P< 0.01; ***P< 0.001.

**Figure S5.** Flow cytometry analysis of MHC-I levels in BMDCs after 0, 12, 24, and 36 h incubation with GSNO (800 μM), as detected by flow cytometry (n=4). The data were shown as mean ± SD. Statistical analysis was performed by One-way ANOVA with a Tukey multiple comparisons test. Other groups were compared with the 0 h group. *P<0.05; **P< 0.01; ***P< 0.001.

**Figure S6.** (a) TEM images of Im-vesicle, NOnanoPL, m-vesicle, nanoPL, m@nanoPL, and Im@NOnanoPL. All images were acquired at high magnification to resolve nanoscale structural features. Scale bar: 50 nm. (b) The corresponding particle size distributions were quantified from TEM images.

**Figure S7.** In vitro biocompatibility evaluation of Im@NOnanoPL in DC2.4 cells. a, CCK-8 assay showing the effects of Im@NOnanoPL on the viability of DC2.4 cells. b, Calcein-AM/propidium iodide (PI) staining of DC2.4 cells after treatment with Im@NOnanoPL (100 μM). c, Rhodamine 123 (RH-123) staining to assess mitochondrial membrane potential in DC2.4 cells treated with Im@NOnanoPL (100 μM).

**Figure S8.** Confocal microscopic images of DC2.4 cells after incubating with Im@NOnanoPL and other formulations for 2 h. The nuclei and lysosomes were stained with DAPI (blue) and Lysotracker Green (green), amide-coupled Cy5 labeling for stable cell membrane vesicles surface modification, Scale bar: 10 μm.

**Figure S9.** Evaluation of MHC-I_OVA_ complex levels in BMDCs after 12 and 24 h incubation with Im@NOnanoPL and other formulations. (NOC-18: 100 μM), as detected by flow cytometry (n=4). The data were shown as mean ± SD. Statistical analysis was performed by One-way ANOVA with a Tukey multiple comparisons test.

**Figure S10.** Representative flow cytometry analysis of CD69^+^ in CD8^+^ CD3^+^ T cells after 24h co-culture, as detected using flow cytometry.

**Figure S11.** Representative flow cytometry analysis of CD103^+^Cy5.5^+^ or CD8^+^Cy5.5^+^ on CD11c^+^ DCs in lymph nodes after 12 h treatment with Cy5.5 labeled Im@NOnanoPL.

**Figure S12.** Biodistribution of Im@NOnanoPL nanovaccine following subcutaneous administration. a, C57BL/6N mice were subcutaneously injected with Cy5.5-labeled Im@NOnanoPL nanovaccine (Cy5.5 concentration: 3 µg/mL). At 6 h post-injection, Major organs including heart (H), liver (Li), spleen (Sp), lung (Lu), and kidney (Ki), as well as the draining lymph nodes were harvested for ex vivo fluorescence imaging using an IVIS system. b, Quantification of total fluorescence intensity in the heart, liver, spleen, lung, and kidney. Data are presented as mean ± s.d. (n = 4). P values were determined using one-way ANOVA followed by Tukey’s post hoc test.

**Figure S13.** Lymph node size of mice after different treatments (G1: PBS, G2: m@nanoPL, G3: Im@nanoPL, G4: m@NOnanoPL, G5: Im@NOnanoPL), (n = 3).

**Figure S14.** Representative flow cytometry analysis of MHC-I_OVA_ complex levels on DCs in lymph nodes under different treatments.

**Figure S15.** Representative flow cytometry analysis of GZMB ^+^ among CD8^+^ T cells in lymph nodes under different treatments.

**Figure S16.** Representative flow cytometry analysis of IFN-γ^+^ among CD8^+^ T cells in lymph nodes under different treatments.

**Figure S17.** Quantification of immune responses of CD4^+^ T cells in the lymph nodes as measured by the percentage of GZMB ^+^ among CD4^+^ T cells (n = 4).

**Figure S18.** Quantification of immune responses of CD4^+^ T cells in the lymph nodes as measured by the percentage of IFN-γ^+^ among CD4^+^ T cells.

**Figure S19.** Flow cytometry analysis of IFN-γ and granzyme B expression in CD4⁺ and CD8⁺ T cells from vaccinated mice after phorbol myristate acetate and ionomycin stimulation (n = 4). Statistical analysis was performed using the one-way ANOVA with Tukey’s multiple comparisons test.

**Figure S20.** Representative flow cytometry images of central T cells and memory T cells in spleen following various treatments in B16-OVA cell pulmonary metastasis model.

**Figure S21.** Body weight changes of the mice after different treatments in MC38 cell tumor model (n = 6 mice per group).

**Figure S22.** Ki67 staining (red) of tumors, Scale bar 200μm (G1: PBS, G2: αPD-1, G3: Im@nanoPL, G4: m@NOnanoPL, G5: Im@NOnanoPL, G6: Im@NOnanoPL+ αPD-1).

**Figure S23.** Representative flow cytometry images of the CD8^+^ or CD4^+^ T cells in the tumors following various treatments in MC38 cell tumor model.

**Figure S24.** Representative flow cytometry images of the CD86^+^ F4/80^+^ macrophages in the tumors following various treatments in MC38 cell tumor model.

**Figure S25.** Representative flow cytometry images of the CD206^+^ F4/80^+^ macrophages in the tumors following various treatments in MC38 cell tumor model.

**Figure S26.** Representative flow cytometry images of the NK1.1^+^CD11b^+^ cells in the tumors following various treatments in MC38 cell tumor model.

**Figure S27.** Representative flow cytometry images of the CD80^+^CD86^+^ DCs in the tumors following various treatments in MC38 cell tumor model.

**Figure S28.** Percentage of CD69^+^ CD4^+^ T cells in spleen of MC38-OVA tumor mouse after 24h co-culture with OVA polypeptide, as detected using flow cytometry (n= 4). The data were shown as mean ± SD. Statistical analysis was performed by One-way ANOVA with a Tukey multiple comparisons test.

**Figure S29.** Flow cytometry analysis of granzyme B expression on CD4⁺ T cells in spleen of MC38-OVA tumor mouse after 24h co-culture with OVA_257–264_ (n= 4). The data were shown as mean ± SD. Statistical analysis was performed by One-way ANOVA with a Tukey multiple comparisons test.

**Figure S30.** Hemolysis assay of Im@NOnanoPL. Representative images of red blood cell suspensions incubated with Im@NOnanoPL at different concentrations, with Saline and deionized water serving as negative and positive controls, respectively (left). Quantification of hemolysis percentages (right). Data are shown as mean ± s.d. (n = 3).

**Figure S31.** Routine blood tests and statistical analysis of mice in different treatment groups, data were shown as mean ± s.e.m, n = 4.

**Fig. S32.** H&E staining of major organs collected from C57BL/6N mice bearing MC38 tumors. MC38 tumor of different groups at Day 18. Scale bar, 100 µm. G1: PBS, G2: αPD-1, G3: Im@nanoPL, G4: m@NOnanoPL, G5: Im@NOnanoPL, G6: Im@NOnanoPL+ αPD-1.

**Table S1. Benchmarking of Im@NOnanoPL against Conventional STING Agonists**

| Feature | Conventional STING Agonists (e.g., CDNs) | Im@NOnanoPL (NO-based Platform) |
| --- | --- | --- |
| Potency | High potency in IFN-I induction but often leads to systemic "cytokine storm" or T-cell ablation at high doses. | Dual potency: NO activates the STING-TBK1-IRF3 axis while uniquely upregulating MHC-I to enhance cross-presentation. |
| Safety | High risk of systemic toxicity; poor metabolic stability in vivo. | Excellent biosafety: No detectable hemolysis or pathological damage to major organs. |
| Delivery | Poor bioavailability; requires complex carriers to prevent rapid clearance or systemic diffusion. | Superior delivery: ICD membranes facilitate lymph node (LN) targeting; gaseous NO is freely diffusible, bypassing DC uptake barriers. |
| Antigen Coverage | Usually limited to specific peptides or a few neoantigens. | Broad-spectrum: ICD membranes provide a full repertoire of endogenous antigens, overcoming tumor heterogeneity. |
